# Artificial Seawater Models Affect Sorption of Adenine and Related Molecules Sorption onto Montmorillonite: Implications for Early Mars and Earth Oceans

**DOI:** 10.1021/acsomega.5c03351

**Published:** 2025-06-26

**Authors:** Giulio Wilgner Ferreira, Bruno Estevam Pintor, Rafael Block Samulewski, Dimas Augusto Morozin Zaia

**Affiliations:** † Laboratório de Química Prebiótica-LQP, Departamento de Química, 37894 Universidade Estadual de Londrina, 86057-970 Londrina, PR, Brazil; ‡ Programa de Pós-Graduação em Ciência e Engenharia de Materiais (PPGCEM) - Universidade Tecnológica Federal do Paraná UTFPR Campus Apucarana 86812-460 Apucarana, PR , Brazil

## Abstract

The adsorption of biomolecules onto mineral surfaces plays a crucial role in the context of prebiotic chemistry, as originally proposed by John Desmond Bernal. According to Bernal’s hypothesis, clay minerals could have facilitated the concentration and stabilization of organic molecules, creating favorable microenvironments for prebiotic reactions and polymerization processes. In this study, we evaluate the adsorption behavior of adenine, adenosine, and adenosine monophosphate (AMP) onto montmorillonite under different artificial seawater conditions, simulating early Earth and early Mars aqueous environments. The adsorption efficiency varied depending on the ionic composition of the solution, with AMP exhibiting the highest adsorption, likely due to its phosphate group interacting with divalent cations in solution and clay surfaces. These findings suggest that montmorillonite could have played a significant role in the retention of organic molecules under prebiotic conditions. In particular, these results reinforce the idea that early oceans, lakes, or hydrothermal systems with high mineral content might have acted as selective reservoirs for prebiotic compounds. These insights contribute to our understanding of how prebiotic chemistry could have evolved in different planetary scenarios, with implications for the origin of life on Earth and potentially on Mars.

## Graphical abstract

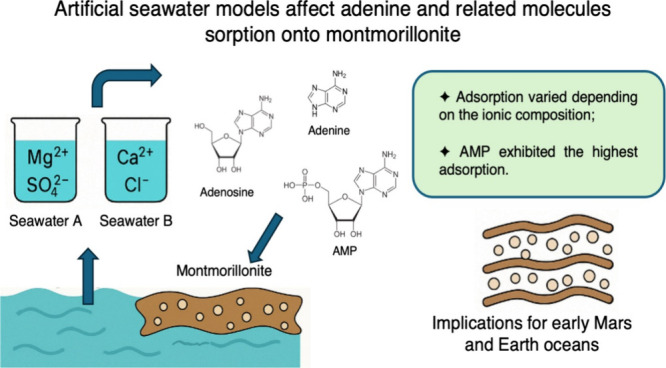

## 1 Introduction

Before the origin of life, the emerged land on Earth was only 12% of today’s emerged land, meaning that oceans covered practically the entire planet.^1^ Therefore, all molecules or precursor molecules delivered to the planet by meteorites, comets, or interplanetary dust or even molecules synthesized in the planet fell in this ocean, and would be diluted, which would make molecular evolution impossible.^2,3^ Therefore, among Bernal’s suggestions for the origin of life, preconcentration of biomolecules and precursors of biomolecules is the most important step.^4^ There are several ways to preconcentrate biomolecules: sorption, wetting/drying cycles, freezing/sublimation, and sorption/precipitation with minerals.

Nucleic acid bases, nucleosides, and nucleotides could be synthesized on the prebiotic Earth, delivered to Earth by meteorites or even synthesized in experiments simulating the interstellar medium.^5–16^ In addition, approximately 4.55–4.00 billion years ago, montmorillonite existed on the prebiotic Earth^17^ and was also detected on the surface of Mars.^18^ As nucleic acid bases, nucleosides, and nucleotides, as well as montmorillonite existed on the prebiotic Earth, the interaction between them is an important issue for prebiotic chemistry.

In the current work adenine, adenosine, and AMP (Figure 1) were dissolved in water and in two different seawaters. Our group suggested the two seawaters used in the current work; the first was named seawater-A and contains high Mg^2+^ and SO_4_^2–^ concentrations,^19^ and the second was named seawater-B, and contains high Ca^2+^ and Cl^–^ concentrations.^20^ Seawater-A and seawater-B were suggested based on the work of Izawa et al.^21^ and Halevy and Bachan,^22^ respectively. In the Section 5, we will explain why these seawaters could be used as models for the prebiotic Earth, Mars, and moons of the Solar System.

**Figure 1:**
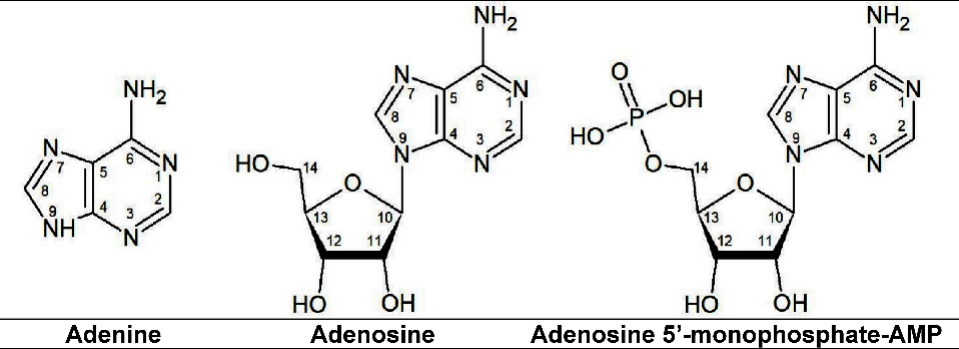
Molecular structures of adenine, adenosine, and adenosine 5′ monophosphate.

The adsorption of adenine, adenosine, and AMP onto minerals has been studied by several research groups (Table S1). The data presented in Table S1 are not intended to be a review on the adsorption of nucleic acid bases, nucleosides, and nucleotides onto minerals, but provide a sample of the work carried out to date. The data will be used to explain the importance of the conditions used in the experiments, which aim to resemble those on the prebiotic Earth, meaning before life existed on our planet. Our focus will be on the solutions used to dissolve the nucleic acid bases, nucleosides, and nucleotides. The majority of the works presented in Table S1 were not carried out under conditions that existed on the Earth before the origin of life. However, this issue will be discussed in the Section 5.

In the present work, adsorption isotherms of adenine, adenosine, and AMP adsorbed onto montmorillonite were obtained. These molecules were dissolved in water, artificial seawater-A (high Mg^2+^ and SO_4_^2–^ concentrations), and artificial seawater-B (high Ca^2+^ and Cl^–^ concentrations). The *q*_max_ (the theoretical limit of adsorption), *n* (heterogeneity factor), *R*_L_ (separation factor), Δ*S* (adsorption entropy), Δ*H* (adsorption enthalpy), and Δ*G* (adsorption Gibbs free energy) were also obtained. The effect of the pH on the adsorption of adenine, adenosine, and AMP onto montmorillonite were studied, as well as the interaction between these molecules and montmorillonite, using FT-IR spectroscopy. Desorption experiments of adenine, adenosine, and AMP from montmorillonite using calcium chloride were carried out.

## 2 Materials and Methods

### 2.1 Materials

Ultrapure water was obtained from Simplicity Merck with 18.0 MΩ cm at 25 °C.

#### 2.1.1 Reagents

All reagents used in the experiments were analytical grade PA.

Montmorillonite-KSF (SiO_2_ = 54%, Al_2_O_3_ = 17%, Fe_2_O_3_ = 5.2%, CaO = 1.5%, MgO = 2.5%, Na_2_O = 0.4%, and K_2_O = 1.5%) was purchased from Acros Organics and used as received. Montmorillonite KSF was selected for due to its well-characterized surface chemistry and its frequent use in adsorption studies. Although natural montmorillonites are available and have been used in prebiotic chemistry studies, KSF provides a reproducible and well-controlled model for adsorption experiments. Previous studies have demonstrated that KSF retains key physicochemical properties relevant to prebiotic adsorption studies.^23–28^

Adenine, adenosine, and adenosine 5′-monophophate-AMP were purchased from Sigma-Aldrich and used as received (Figure 1).

#### 2.1.2 Artificial Seawater

Artificial seawater-A was prepared as described by Zaia.^19^ The following salts were dissolved in 1.0 L of ultrapure water: Na_2_SO_4_ (0.271 g), MgCl_2_·6H_2_O (0.500 g), CaCl_2_·2H_2_O (2.50 g), KBr (0.050 g), K_2_SO_4_ (0.400 g), and MgSO_4_ (15.00 g). Each salt was dissolved in the order they are shown.

Artificial seawater-B was prepared as described by Samulewski et al.^20^ The following salts were dissolved in 1.0 L of ultrapure water: MgCl_2_ (0.950 g), CaCl_2_·2H_2_O (29.400 g), KCl (1.490 g), NaCl (1.170 g).

### 2.2 Methods

#### 2.2.1 Determination of pH at Point of Zero Charge (pHpzc)

The point of zero charge (pH_PZC_) of montmorillonite was determined as described by Uehara.^29^ In two 2.0 mL Eppendorf tubes, 50 mg of mineral samples were added, followed by 125 μL of 1.0 mol L^–1^ KCl solution added to one tube and 125 μL ultrapure water to the other. Both tubes were shaken for 30 min, and after 24 h, the pH was measured. The pH_PZC_ was calculated using eq 1:

$${\mathrm{pH}}_{\mathrm{PZC}}=2{\mathrm{pH}}_{\mathrm{KCl}(1.0\mathrm{mol}/L)}-{\mathrm{pH}}_{\mathrm{ultrapure}\;\mathrm{water}}$$ 1

#### 2.2.2 UV Spectroscopy

The absorbance of adenine, adenosine, and adenosine 5′-monophophate-AMP was measured at 260 nm by spectrophotometry (Spectronic Genesys). To calculate the amount of analyte absorbed onto the clay, eq 2 was used:

$$\left(C_{\mathrm{adsorbed}}/\mathrm{\mu g})=(C_{\mathrm{initial}}-C_{\mathrm{solution}}\right)$$ 2

where *C*_solution_ = (*C*_initial_) (Abs_sample_/Abs_initial_).

#### 2.2.3 Effect of pH on the Adsorption of the Molecules

The concentration of the analytes (adenine, adenosine, and adenosine 5′-monophophate-AMP) was 800 mg L^–1^. They were dissolved in ultrapure water, artificial seawater-A (high Mg^2+^ and SO_4_^2–^ concentrations), or artificial seawater-B (high Ca^2+^ and Cl^–^ concentrations). Then, approximately 50 mg of montmorillonite and 5 mL of adenine, adenosine, and adenosine 5′-monophosphate solutions were added to a conical tube (15 mL). The pH of the solutions was adjusted with sodium hydroxide (0.10 mol L^–1^) or hydrochloric acid (0.10 mol L^–1^) from 2.0 to 11.0. The suspensions were stirred for 24 h at 25 °C and the samples were then centrifuged at 9000 rpm for 10 min. The supernatant was used to determine the adenine, adenosine, and adenosine 5′-monophosphate.

#### 2.2.4 Adsorption Isotherm

Montmorillonite (10 mg) and 1.0 mL of adenine, adenosine, and adenosine 5′-monophosphate dissolved in ultrapure water, seawater-A (high Mg^2+^ and SO_4_^2–^ concentrations), or artificial seawater-B (high Ca^2+^ and Cl^–^ concentrations) in the following concentrations: 20, 40, 80, 100, 150, 200, 300, 400, 500, 600, 650, 780, and 800 mg L^–1^ were added to Eppendorf tubes (2 mL). Each isotherm was performed in triplicate. The pH of the solutions was adjusted to 5.00 with sodium hydroxide or hydrochloric acid 0.10 mol L^–1^. The suspensions were stirred for 24 h at 20, 35, and 50 °C. The temperature during the isotherm experiments was maintained stable for 24 h at respective temperatures using a thermostatic bath and continuous monitoring to avoid fluctuations that could affect the adsorption equilibrium. The samples were centrifuged at 9000 rpm for 10 min. The supernatant was used to determine the adenine, adenosine or adenosine 5′-monophophate-AMP. The mineral was lyophilized and characterized.

In the current work, the results of adenine, adenosine, and adenosine 5′-monophosphate adsorptions onto montmorillonite were fitted to nonlinear isotherm models of Langmuir, Freundlich, and SIPs to verify which model presented the best adjustment.

The nonlinear expression of the Langmuir isotherm model (eq 3).^30,31^

$$\theta =\frac{k_{\mathrm{eq}}bC}{\left(1+C\right)}$$ 3

Where *C* (mg L^–1^) is the concentration of adenine, adenosine, or adenosine 5′-monophophate-AMP in the solution after the equilibrium, θ (mg g^–1^) is the amount of adenine, adenosine, or adenosine 5′-monophophate-AMP adsorbed onto montmorillonite (difference between initial concentrations and the concentration after the equilibrium), *b* (mg g^–1^) is the theoretical limit of adsorbed adenine, adenosine, or adenosine 5′-monophophate-AMP onto montmorillonite, and *k*_eq_ (L mg^–1^) is equilibrium constant (adsorbate–adsorbent).

The nonlinear expression of the Freundlich isotherm model (eq 4)^30,31^

$$\theta =K_{f}C^{n}$$ 4

where *C* (mg L^–1^) is the concentration of adenine, adenosine, or adenosine 5′-monophophate-AMP in the solution after the equilibrium, θ (mg g^–1^) is the amount of adenine, adenosine, or adenosine 5′-monophophate-AMP adsorbed onto montmorillonite (difference between initial concentrations and concentration after the equilibrium), and *K*_f_ and *n* are empirical constants.

The nonlinear expression of the SIPs isotherm model (eq 5)^30,31^

$$\theta =\frac{b(KC{)}^{1/n}}{1+(KC{)}^{1/n}}$$ 5

where *C* (mg L^–1^) is the concentration of adenine, adenosine, or adenosine 5′-monophophate-AMP in the solution after the equilibrium, θ (mg g^–1^) is the amount of adenine, adenosine, or adenosine 5′-monophophate-AMP adsorbed onto montmorillonite (difference between initial concentrations and concentration after the equilibrium), *b* (mg g^–1^) is the theoretical limit of adsorbed adenine, adenosine, or adenosine 5′-monophophate-AMP onto montmorillonite, and *K* and *n* are empirical constants.

#### 2.2.5 Desorption

After the adsorption experiments, the desorption experiments were carried out using CaCl_2_ solution (0.1 mol L^–1^). The desorption experiments were repeated 3 times. In each tube containing 50 mg of analyte adsorbed onto the mineral, 3.0 mL of the CaCl_2_ solution were added.^32,33^ Each desorption step was carried out over a period of 24 h. After this period, the supernatants were removed and quantified by UV spectrophotometry. This process was repeated 2 more times. At the end, the desorption of each step was added and the total desorption for each analyte was obtained.

#### 2.2.6 Fourier Transform Infrared Spectroscopy-FTIR

Montmorillonite samples were analyzed in ATR-FTIR. The spectra were obtained with a resolution of 4 cm^–1^ in the range 4000–400 cm^–1^ in Bruker-Vertex 70 spectrometer equipped with ATR accessory with a Ge Crystal 45°.

## 3 Results

### 3.1 Effect of pH on the Adsorption of the Molecules

Adsorption of adenine, dissolved in ultrapure water, onto montmorillonite decreased with the increase in pH. The adsorption of adenosine 5′-monophophate-AMP, dissolved in ultrapure water, increased until the pH reached 5.0 and then decreased. Adsorption of adenosine decreased until the pH reached 5.0 and then increased again (Figure 2A).

**Figure 2:**
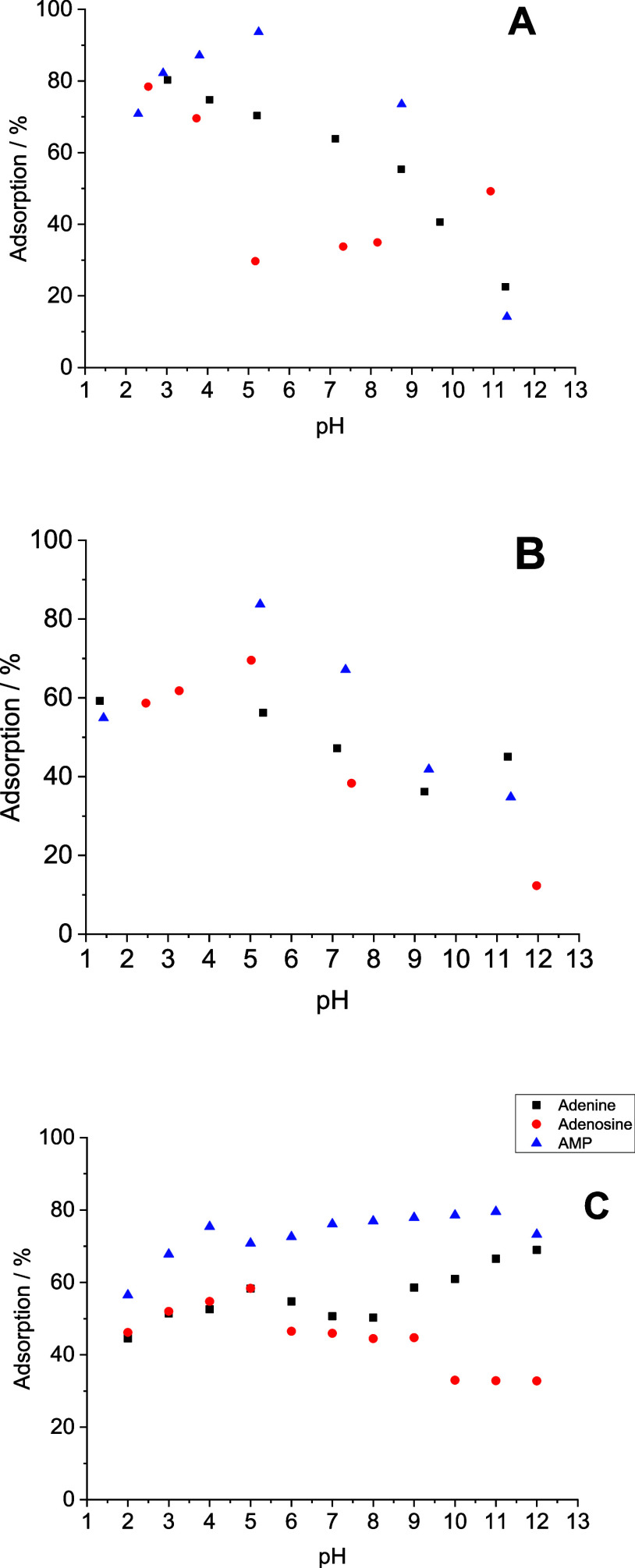
Effect of the pH on the adsorption of adenine (800 mg L^–1^), adenosine (800 mg L^–1^), and AMP- adenosine 5′-monophosphate (800 mg L^–1^) onto montmorillonite (50 mg). Adenine, adenosine and AMP- adenosine 5′-monophosphate were dissolved in (A) ultrapure water, (B) artificial seawater-A (high Mg^2+^ and SO_4_^2–^ concentrations), and (C) artificial seawater-B (high Ca^2+^ and Cl^–^ concentrations). Each value is mean of three experiments. The samples were stirred for 24 h at 25 °C. Artificial seawater-A (high Mg^2+^ and SO_4_^2–^ concentrations) and artificial seawater-B (high Ca^2+^ and Cl^–^ concentrations) were prepared as described by Zaia^19^ and Samulewski et al.^20^ respectively.

In addition, dissolving these molecules in artificial seawater-A (high Mg^2+^ and SO_4_^2–^ concentrations), the adsorption of AMP and adenosine increased until the pH reached 6.0 and then decreased. However, from pH 6.0 to pH 10, the adsorption of adenine decreased, and then increased (Figure 2B).

Finally, another trend was observed dissolving these molecules in artificial seawater-B (high Ca^2+^ and Cl^–^ concentrations) (Figure 2C). For adenine, adsorption decreased from pH 6.0 to pH 8.0 and then increased (Figure 2C). For adenosine, adsorption decreased in pH 6.0, remained constant until pH 9.0, before decreasing again at pH 10, and remaining constant until the pH reached 12.0 (Figure 2C). AMP presented a slight decrease in the adsorption at around pH 6.0 and then adsorption increased with increasing pH (Figure 2C).

### 3.2 Adsorption Isotherm of the Molecules

In general, nonlinear isotherm fits showed better *R* ^2^/RMSE parameters for the Langmuir and SIPs models than for the Freundlich model (Table 1). In addition, in general, for all adenine and AMP samples, the *R* ^2^/RMSE parameters for the SIPs model was slightly better than the Langmuir model (Table 1). An inverse trend was observed for the adenosine samples (Table 1).

**Table 1:** Parameters of Nonlinear Adsorption Models for the Samples of Adenine, Adenosine, and Adenosine 5′-Monophosphate Adsorbed onto Montmorillonite at Different Temperatures^a^

			**parameters**				
**solution**	***T*/°C**	**model**	***K***	***q*_max_**	***n***	***R*** ^**2**^	**RMSE**
adenine							
ultrapure water	20	Langmuir	0.03958	35.5		0.9970	0.5922
		Freundlich	12.9		0.1618	0.9758	1.677
		SIPs	0.03529	10.73	1.482	0.9999	0.0814
	35	Langmuir	0.1091	37.5		0.9901	1.548
		Freundlich	16.62		0.1398	0.9939	1.208
		SIPs	0.07643	39.42	0.6226	0.9968	0.8722
	50	Langmuir	0.2337	38.94		0.9819	1.798
		Freundlich	23.55		0.0898	0.9990	0.4313
		SIPs	0.05856	47.41	0.4216	0.9982	0.5639
ASW-A	20	Langmuir	0.2038	26.25		0.9968	0.5106
		Freundlich	15.37		0.0905	0.9864	1.057
		SIPs	0.2341	26.72	0.8065	0.9969	0.5032
	35	Langmuir	0.1186	28.34		0.9957	0.6226
		Freundlich	15.55		0.1008	0.9943	0.7186
		SIPs	0.1602	29.53	0.5681	0.9980	0.4302
	50	Langmuir	0.1871	33.37		0.9976	0.5476
		Freundlich	24.27		0.05263	0.9929	0.9505
		SIPs	0.08827	32.97	1.872	0.9982	0.4796
ASW-B	20	Langmuir	0.0355	56.15		0.9780	3.091
		Freundlich	6.644		0.3706	0.9017	6.537
		SIPs	0.0532	49.89	1.628	0.9898	2.103
	35	Langmuir	0.1036	63.13		0.7256	12.8
		Freundlich	17.75		0.2212	0.7172	13.00
		SIPs	0.0605	72.71	0.6048	0.7023	8.34
	50	Langmuir	0.06012	81.09		0.9229	8.554
		Freundlich	15.23		0.2862	0.8333	12.57
		SIPs	0.08069	75.98	1.431	0.9253	8.42
adenosine							
ultrapure water	20	Langmuir	0.05368	31.32		0.9976	0.4690
		Freundlich	17.53		0.0917	0.9972	0.5087
		SIPs	0.02867	30.01	1.813	0.9973	0.4957
	35	Langmuir	0.01795	48.85		0.9923	1.143
		Freundlich	10.59		0.2393	0.9903	1.278
		SIPs	0.01795	48.83	1.001	0.9910	1.235
	50	Langmuir	0.02503	54.58		0.9955	0.9951
		Freundlich	15.62		0.200	0.9919	1.337
		SIPs	0.02176	51.86	1.603	0.9958	0.9654
ASW-A	20	Langmuir	0.0729	44.05		0.9985	0.5284
		Freundlich	21.79		0.1179	0.9973	0.7047
		SIPs	0.08945	46.52	0.7023	0.9986	0.5051
	35	Langmuir	0.02991	61.58		0.9893	1.909
		Freundlich	15.07		0.2314	0.9611	3.639
		SIPs	0.03065	56.16	1.603	0.9946	1.351
	50	Langmuir	0.02588	75.33		0.9691	3.899
		Freundlich	13.72		0.2862	0.9579	4.554
		SIPs	0.02722	73.19	1.085	0.9641	4.205
ASW-B	20	Langmuir	0.01375	62.23		0.9803	2.719
		Freundlich	5.762		0.3696	0.9317	5.067
		SIPs	0.01489	60.20	1.085	0.9788	2.820
	35	Langmuir	0.006665	90.41		0.9760	3.80
		Freundlich	3.34		0.5005	0.9505	5.46
		SIPs	0.01274	73.42	1.229	0.9736	3.985
	50	Langmuir	0.006521	110.7		0.9798	4.026
		Freundlich	3.301		0.5392	0.9442	6.701
		SIPs	0.01092	87.39	1.432	0.9877	3.146
adenosine 5′-monophosphate-AMP							
ultrapure water	20	Langmuir	0.2687	64.03		0.9997	0.3671
		Freundlich	45.8		0.06385	0.9972	1.119
		SIPs	0.1985	63.09	1.277	0.9998	0.3003
	35	Langmuir	0.09614	90.3		0.9969	1.533
		Freundlich	32.1		0.2062	0.9753	4.347
		SIPs	0.09622	85.42	1.297	0.9983	1.146
	50	Langmuir	0.09272	105.1		0.9977	1.537
		Freundlich	26.37		0.307	0.9731	5.215
		SIPs	0.1061	96.64	1.284	0.9996	0.6071
ASW-A	20	Langmuir	0.08508	62.15		0.9967	1.092
		Freundlich	23.10		0.1856	0.9698	3.288
		SIPs	0.0842	59.52	1.26	0.9979	0.864
	35	Langmuir	0.06675	74.82		0.9864	2.64
		Freundlich	21.32		0.2369	0.9339	5.825
		SIPs	0.07092	67.68	1.647	0.9990	0.712
	50	Langmuir	0.1513	82.79		0.9967	1.436
		Freundlich	35.43		0.1781	0.9813	3.416
		SIPs	0.1419	78.78	1.359	0.9979	1.153
ASW-B	20	Langmuir	0.02721	113.8		0.9311	9.861
		Freundlich	11.31		0.3996	0.8574	14.19
		SIPs	0.03732	105.78	1.307	0.9309	9.875
	35	Langmuir	0.01305	135.8		0.9635	7.391
		Freundlich	6.132		0.5203	0.9036	12.02
		SIPs	0.02502	103.58	1.714	0.9873	4.358
	50	Langmuir	0.01052	147.1		0.9335	10.4
		Freundlich	4.632		0.577	0.9044	12.48
		SIPs	0.02608	101.76	2.019	0.9500	9.025

a Each value is mean of three experiments. *k* = constant of Langmuir or adsorbate–adsorbent affinities for Freundlich or SIPS, *q*_max_ = the theoretical limit of adsorbed for adenine, adenosine or adenosine 5′-monophophate, *n* = Freundlich heterogeneity factor, *R* ^2^/RMSE = coefficient of determination/Root Mean Squared Error. The samples were stirred for 24 h at pH 5.00. For all experiments, in Eppendorf tubes, was added 10.0 mg of montmorillonite plus 1.0 mL of adenine, adenosine or adenosine 5′-monophosphate dissolved in ultrapure water (from 20 to 800 mg L^–1^) or ASW-A = artificial sweater-A (from 20 to 800 mg L^–1^) or ASW-B = artificial seawater-B (from 20 to 800 mg L^–1^). Artificial seawater-A (high Mg^2+^ and SO_4_^2–^ concentrations) and artificial seawater-B (high Ca^2+^ and Cl^–^ concentrations) were prepared as described by Zaia^19^ and Samulewski et al.,^20^ respectively.

Among the molecules studied herein (adenine, adenosine, AMP), AMP presented the highest adsorption onto montmorillonite, followed by adenosine and adenine (Table 1). For adenine, adenosine, and AMP, the *q*_max_ presented the highest values when they were dissolved in artificial seawater-B (high Ca^2+^ and Cl^–^ concentrations) (Table 1). For adenine and AMP, the *q*_max_ presented the lowest values when they were dissolved in seawater-A (high Mg^2+^ and SO_4_^2–^ concentrations) (Table 1). Adenosine presented a different trend, the lowest values of *q*_max_ were obtained when dissolving it in ultrapure water (Table 1). With one exception, AMP dissolved in artificial seawater-B (high Ca^2+^ and Cl^–^ concentrations), for all other values, the *q*_max_ presented an increase, when the temperature increased (Table 1), so the adsorption process is endothermic.

The *n* values obtained from the Freundlich isotherm model will not be considered because this model did not present good *R* ^2^/RMSE values (Table 1). For adenine, the effect of the temperature on the *n* values did not show a trend (Table 1). For adenosine and AMP, all *n* values were higher than 1.0, with the exception of adenosine dissolved in artificial seawater-A (high Mg^2+^ and SO_4_^2–^ concentrations) at a temperature of 20 °C. Furthermore, when dissolving adenosine and AMP in artificial seawater-B (high Ca^2+^ and Cl^–^ concentrations) the *n* values increased with an increase in the temperature (Table 1). However, when dissolving adenosine and AMP in ultrapure water and artificial seawater-A (high Mg^2+^ and SO_4_^2–^ concentrations), the effect of the temperature on *n* did not show a trend.

The separation factor (*R*_L_) can be obtained from the Langmuir isotherm:

$$R_{L}=\frac{1}{1+K_{\mathrm{eq}}C_{0}}$$ 6

All *R*_L_ values were lower than 1, indicating a process favorable to adsorption (Table 2). In addition, the *R*_L_ values increased when the adenine, adenosine, and AMP concentrations decreased. Therefore, the adsorption of adenine, adenosine, and AMP became less favorable at low concentrations and more favorable at high concentrations. (Table 2).

**Table 2:** Separation Factor (*R*_L_) for Initial Adenine, Adenosine, or Adenosine 5′-Monophosphate Concentration (*C*_0_) of Isotherm Samples at Different Temperatures

	**ultrapure water**			**artificial seawater-A**			**artificial seawater-B**		
*C*_0_/mg L^–1^	**20 °C**	**35 °C**	**50 °C**	**20 °C**	**35 °C**	**50 °C**	**20 °C**	**35 °C**	**50 °C**
adenine									
20	0.5582	0.3143	0.1762	0.1970	0.2966	0.2109	0.5848	0.3255	0.4541
40	0.3871	0.1864	0.0966	0.1093	0.1741	0.1179	0.4132	0.1944	0.2937
80	0.2400	0.1028	0.0508	0.0578	0.0953	0.0626	0.2604	0.1077	0.1721
100	0.2017	0.0840	0.0410	0.0468	0.0778	0.0507	0.2198	0.0880	0.1426
150	0.1442	0.0576	0.0277	0.0317	0.0532	0.0344	0.1581	0.0605	0.0998
200	0.1122	0.0438	0.0209	0.0239	0.0405	0.0260	0.1235	0.0460	0.0768
300	0.0777	0.0296	0.0141	0.0161	0.0273	0.0175	0.0858	0.0312	0.0525
400	0.0594	0.0224	0.0106	0.0121	0.0206	0.0132	0.0658	0.0236	0.0399
500	0.0481	0.0180	0.0085	0.0097	0.0166	0.0106	0.0533	0.0189	0.0322
600	0.0404	0.0150	0.0071	0.0081	0.0139	0.0088	0.0448	0.0158	0.0270
650	0.0374	0.0139	0.0065	0.0075	0.0128	0.0082	0.0415	0.0146	0.0250
780	0.0314	0.0116	0.0055	0.0063	0.0107	0.0068	0.0349	0.0122	0.0209
800	0.0306	0.0113	0.0053	0.0061	0.0104	0.0066	0.0340	0.0119	0.0204
adenosine									
20	0.4823	0.7358	0.6664	0.4068	0.6257	0.6589	0.7843	0.8825	0.8846
40	0.3177	0.5821	0.4997	0.2554	0.4553	0.4914	0.6452	0.7896	0.7931
80	0.1889	0.4105	0.3331	0.1464	0.2947	0.3257	0.4762	0.6524	0.6572
100	0.1570	0.3578	0.2855	0.1206	0.2506	0.2787	0.4211	0.6002	0.6053
150	0.1105	0.2708	0.2103	0.0838	0.1823	0.2048	0.3265	0.5003	0.5055
200	0.0852	0.2179	0.1665	0.0642	0.1432	0.1619	0.2667	0.4288	0.4340
300	0.0585	0.1566	0.1175	0.0437	0.1003	0.1141	0.1951	0.3336	0.3383
400	0.0445	0.1222	0.0908	0.0332	0.0771	0.0881	0.1538	0.2729	0.2771
500	0.0359	0.1003	0.0740	0.0267	0.0627	0.0717	0.1270	0.2309	0.2347
600	0.0301	0.0850	0.0624	0.0224	0.0528	0.0605	0.1081	0.2002	0.2036
650	0.0279	0.0789	0.0579	0.0207	0.0489	0.0561	0.1006	0.1877	0.1909
780	0.0233	0.0667	0.0487	0.0173	0.0411	0.0472	0.0853	0.1614	0.1643
800	0.0228	0.0651	0.0476	0.0169	0.0401	0.0461	0.0833	0.1580	0.1609
adenosine 5′-monophosphate-AMP									
20	0.1569	0.3421	0.3503	0.3702	0.4283	0.2484	0.6476	0.7930	0.8262
40	0.0851	0.2064	0.2124	0.2271	0.2725	0.1418	0.4788	0.6570	0.7038
80	0.0445	0.1151	0.1188	0.1281	0.1577	0.0763	0.3148	0.4892	0.5430
100	0.0359	0.0942	0.0974	0.1052	0.1303	0.0620	0.2687	0.4338	0.4873
150	0.0242	0.0648	0.0671	0.0727	0.0908	0.0422	0.1968	0.3381	0.3879
200	0.0183	0.0494	0.0512	0.0555	0.0697	0.0320	0.1552	0.2770	0.3222
300	0.0123	0.0335	0.0347	0.0377	0.0476	0.0216	0.1091	0.2035	0.2406
400	0.0092	0.0253	0.0263	0.0285	0.0361	0.0163	0.0841	0.1608	0.1920
500	0.0074	0.0204	0.0211	0.0230	0.0291	0.0130	0.0685	0.1329	0.1597
600	0.0062	0.0170	0.0177	0.0192	0.0244	0.0109	0.0577	0.1133	0.1368
650	0.0057	0.0158	0.0163	0.0178	0.0225	0.0101	0.0535	0.1055	0.1276
780	0.0047	0.0132	0.0136	0.0148	0.0188	0.0084	0.0450	0.0895	0.1086
800	0.0306	0.0113	0.0053	0.0061	0.0104	0.0066	0.0340	0.0119	0.0204

For better understanding of the adsorption process, the thermodynamic parameters (Δ*H*, Δ*S*, Δ*G*) were obtained.

Using eq 7, the values of the Gibbs free energy (Δ*G*) were calculated:

$$\Delta G=-RT\ln k_{D}$$ 7

where, *k*_D_ is the thermodynamic equilibrium constant (L g^–1^), and *k*_D_ can be obtained by plotting *q*_e_/*C*_e_ versus *q*_e_ and extrapolating *q*_e_ to zero (Figure S1).

The enthalpy (Δ*H*) and entropy (Δ*S*) were calculated using eq 8:

$$\ln\;k_{D}=-\frac{\Delta H}{RT}+\frac{\Delta S}{R}$$ 8

where, *R* is the universal gas constant (8.314 J mol^–1^ K^–1^) and *T* is the temperature (K).

For all experiments, *K*_eq_ and Δ*G* values were higher than 1 and lower than 0, respectively (Table 3). This is an indication that the process is thermodynamically favorable. Furthermore, for all experiments, with one exception, AMP dissolved in artificial seawater-B (high Ca^2+^ and Cl^–^ concentrations), the adsorption was ruled by entropy (Table 3). Alternatively, dissolving AMP in artificial seawater-B (high Ca^2+^ and Cl^–^ concentrations), the adsorption process onto montmorillonite was ruled by enthalpy and entropy (Table 3). The positive enthalpy values indicate that the process is endothermic (Table 3). It should be noted that ΔS values were highest when adenine and AMP were dissolved in artificial seawater-B (high Ca^2+^ and Cl^–^ concentrations) and ultrapure water, respectively (Table 3). Dissolving adenosine in ultrapure water or artificial seawater-A (high Mg^2+^ and SO_4_^2–^ concentrations), the ΔS values were similar (Table 3). For adenine, the entropy adsorption presented the following trend Δ*S*_ASW‑B_ > Δ*S*_ASW‑A_ > Δ*S*_water_. However, for adenosine and AMP, the entropy adsorption presented an inverse trend Δ*S*_ASW‑B_ < Δ*S*_ASW‑A_ < Δ*S*_water_ (Table 3). In addition, the entropy/enthalpy adsorption values for adenine and adenosine dissolved in artificial seawater-B (high Ca^2+^ and Cl^–^ concentrations) were similar (Table 3).

**Table 3:** Thermodynamic Parameters for the Adsorption of Adenine, Adenosine, or Adenosine 5′-Monophosphate onto Montmorillonite^a^

**solution**	**temperature/°C**	***K***_**eq**_	**Δ*G*/** **kJ mol** ^**–1**^	**Δ*H*/** **kJ mol** ^**–1**^	**Δ*S*/J K**^**–1**^ **mol** ^**–1**^
adenine					
ultrapure water	20	35.69	–8.713	2.167	37.10
	35	37.07	–9.256		
	50	38.75	–9.826		
ASW-A	20	26.22	–7.961	6.378	48.76
	35	28.26	–8.561		
	50	33.37	–9.424		
ASW-B	20	48.33	–9.452	8.567	61.46
	35	57.18	–10.366		
	50	66.97	–11.295		
adenosine					
ultrapure water	20	31.27	–8.390	14.448	78.43
	35	48.40	–9.940		
	50	54.53	–10.743		
ASW-A	20	44.01	–9.223	13.436	77.52
	35	62.17	–10.581		
	50	73.61	–11.549		
ASW-B	20	44.59	–9.255	8.542	60.82
	35	54.93	–10.263		
	50	61.81	–11.079		
adenosine 5′-monophosphate-AMP					
ultrapure water	20	64.06	–10.139	13.349	80.36
	35	90.91	–11.554		
	50	106.80	–12.549		
ASW-A	20	62.57	–10.081	7.397	59.77
	35	76.44	–11.110		
	50	83.07	–11.874		
ASW-B	20	77.34	–10.597	–7.491	10.48
	35	64.10	–10.658		
	50	58.07	–10.912		

a The samples were stirred for 24 h at pH 5.00. ASW-A = Artificial seawater-A (high Mg^2+^ and SO_4_^2–^ concentrations) and ASW-B = artificial seawater-B (high Ca^2+^ and Cl^–^ concentrations) were prepared as described by Zaia^19^ and Samulewski et al.,^20^ respectively.

### 3.3 Desorption of the Molecules

For all AMP and adenine samples dissolved in ultrapure water or artificial seawater-A (high Mg^2+^ and SO_4_^2–^ concentrations) samples, the desorption from montmorillonite was in the range of 20 to 12% (Table 4). However, for all samples of adenosine and for adenine dissolved in artificial seawater-B (high Ca^2+^ and Cl^–^ concentrations), the desorption was in the range of 43 to 56%. Finally, for AMP dissolved in artificial seawater-B (high Ca^2+^ and Cl^–^ concentrations), the desorption was approximately 15% (Table 4).

**Table 4:** Desorption of Adenine, Adenosine, and AMP from Montmorillonite Using CaCl_2_ Solution^a^

		**desorption (%)**				
**molecule**	**solvent of molecules**	**1st step**	**2nd step**	**3rd step**	**total (%)**	**average desorption (%)**
adenine	ultrapure water	11.31	4.95	3.07	19.32	6.44
	ASW-A	4.60	4.74	2.51	11.85	3.95
	ASW-B	19.99	13.02	9.50	42.52	14.17
adenosine	ultrapure water	24.39	18.40	13.58	56.38	18.79
	ASW-A	21.79	15.11	11.04	47.94	15.98
	ASW-B	16.83	13.14	9.81	39.78	13.26
AMP	ultrapure water	7.28	5.73	6.14	19.15	6.38
	ASW-A	5.32	5.06	5.31	15.68	5.23
	ASW-B	4.93	5.57	4.82	15.32	5.11

a Each desorption was performed with 3.0 mL of CaCl_2_ solution (0.1 mol L^–1^). ASW-A = Artificial seawater-A (high Mg^2+^ and SO_4_^2–^ concentrations) and ASW-B = artificial seawater-B (high Ca^2+^ and Cl^–^ concentrations) were prepared as described by Zaia^19^ and Samulewski et al.,^20^ respectively.

### 3.4 FT-IR Spectroscopy

The FT-IR spectra of adenine, adenosine, and AMP in the range from 1400 to 1800 cm^–1^ are very similar (Figure S2). For adenine, adenosine, and AMP the band in the region from 1592 to 1602 cm^–1^ could be due to stretching (C_5_–C_6_), stretching (N_3_–C_4_), bending (C_8_–H), and bending (N_9_–H, only for adenine) (Figures 1, S2 and Table 5).^34–37^ After the adsorption of these molecules onto the montmorillonite this band shifted to from 1617 to 1630 cm^–1^ (Table 5, Figure S2). Furthermore, for adenine and adenosine the bands at 1672 and 1662 cm^–1^ due to bending NH_2_, stretching (C_5_–C_6_), and stretching (C_6_–N_1_),^34–37^ shifted to 1685 cm^–1^ (shoulder)/1697 and 1683 cm^–1^, respectively (Table 5 and Figures 1, S2). However, for AMP this band appears at 1693 cm^–1^ and it did shift (Table 5, Figure S2). Finally, due to stretching (C_6_–N_1_), the band at 1640 cm^–1^ vanished (Table 5 and Figures 1, S2).

**Table 5:** Assignments of Frequencies (cm^–1^) in FT-IR Spectra of Montmorillonite, and Adenine, Adenosine, AMP Solid and Adsorbed onto Montmorillonite

**band cm** ^**–1**^					
**mont^a^**	**adenine^b^**	**water^c^**	**ASW-A^d^**	**ASW-B^e^**	**possible assignments**
adenine					
	1600				ν(C_5_–C_6_), ν(N_3_–C_4_), β(C_8_–H), β(N_9_–H)^34–38^
		1623	1624	1619	(C_5_–C_6_), ν(N_3_–C_4_), β(C_8_–H), β(N_9_–H)^34–38^
1630					hydration of the clay^39,40^
	1672				βNH_2_, ν(C_5_–C_6_), ν(C_6_–N_1_)^34–38^
				1685 (shoulder)	
		1700	1697	1697	βNH_2_, ν(C_5_–C_6_), ν(C_6_–N_1_)^34–38^
adenosine					
	1572				
	1602				(C_5_–C_6_), ν(N_3_–C_4_), β(C_8_–H)^34–38^
		1627	1630	1617	(C_5_–C_6_), ν(N_3_–C_4_), β(C_8_–H)^34–38^ or hydration of the clay^39,40^
1630					hydration of the clay^39,40^
	1662				βNH_2_, ν(C_5_–C_6_), ν(C_6_–N_1_)^34–38^
		1693	1693	1683	βNH_2_, ν(C_5_–C_6_), ν(C_6_–N_1_)^34–38^
AMP-adenosine 5′-monophosphate					
	1592				(C_5_–C_6_), ν(N_3_–C_4_), β(C_8_–H)^34–38^
		1628	1629	1618	(C_5_–C_6_), ν(N_3_–C_4_), β(C_8_–H)^34–38^ or hydration of the clay^39,40^
1630					hydration of the clay^39,40^
	1641				ν(C_6_–N_1_)^34–36,38^
	1693	1693	1693	1683	βNH_2_ ^34–36,38^

a Mont = montmorillonite.

b Adenine or adenosine or AMP = solid adenine or solid adenosine or solid AMP.

c Water = adenine or adenosine or AMP dissolved in ultrapure water.

d ASW-A = adenine or adenosine or AMP dissolved in artificial seawater-A.

e ASW-B = adenine or adenosine or AMP dissolved in artificial seawater-B. The samples were shaken by 24 h at pH 5.00. ASW-A = Artificial seawater-A (high Mg^2+^ and SO_4_^2–^ concentrations) and ASW-B = artificial seawater-B (high Ca^2+^ and Cl^–^ concentrations) were prepared as described by Zaia^19^ and Samulewski, et. al,,^20^ respectively. ν = stretching; β = in-plane bending.

## 4 Discussion

### 4.1 Effect of pH on the Adsorption of the Molecules

The adsorption of adenine onto montmorillonite presented a similar trend when dissolved in ultrapure water or artificial seawater-A (high Mg^2+^ and SO_4_^2–^ concentrations) (Figure 2A, B). These data could be explained in terms of the electrostatic interaction between positively charged adenine (at acid pH) and negatively charged montmorillonite (Table S2). Several authors also observed the same trend in the adsorption of adenine onto several minerals, such as clays,^14,41–45^ zeolites,^46^ and ferrihydrite.^47^ However, because the adsorption of adenine dissolved in artificial seawater-B (high Ca^2+^ and Cl^–^ concentrations) increased as the pH increased (Figure 2C), it could not be explained as only an electrostatic interaction. It should be noted that the adsorption of adenine onto rutile and allophone presented the same outcome.^48,49^ However, the authors attributed the increase in adsorption with increasing pH values to a combination of electrostatic interaction, interaction with metals, hydrogen-bonding, and van der Waals forces.^48,49^ Furthermore, the effect of the pH on the adsorption of adenine onto anatase presented a different trend to rutile.^49,50^ For anatase, the neutral adenine adsorbed more than positively or negatively charged adenine.^50^ In addition, the adenine adsorption onto metal-modified montmorillonite depends on the pH and the metals.^42^ For Co, Ni-montmorillonite, the maximum adsorption occurred at around pH 9.0, and for Cu-montmorillonite, the pH did not have an effect on the adsorption of adenine.^42^ Thus, the adsorption of adenine is due to the interaction with the titanium of anatase and metals of montmorillonite.

When dissolved in the artificial seawaters, adenosine presented the highest adsorption onto montmorillonite at pH 5.0, after which the adsorption decreased as pH increased (Figure 2B,C). However, electrostatic interactions alone cannot explain these data, because at pH 5.0 adenosine is an uncharged molecule (Table S2). Therefore, the effect of the salts of the artificial seawater should be considered. Adenosine also presented the same trend when adsorbed onto montmorillonite and metal-modified montmorillonite^41–44^ and goethite.^51^ The adsorption of adenosine dissolved in water could be explained as an electrostatic interaction until the pH reaches ≈5, (Figure 2A, Table S2). For pH values higher than 5.0, the increase in the adsorption of adenosine onto montmorillonite is not due to electrostatic interaction (Figure 2A, Table S2). Furthermore, the adsorption of adenosine onto allophane and rutile increased as the pH increased.^48,49^

When dissolving AMP in ultrapure water or artificial seawater-A (high Mg^2+^ and SO_4_^2–^ concentrations), the adsorption onto montmorillonite could be explained as an electrostatic interaction between positively charged AMP (at acidic pH) and negatively charged montmorillonite (Figure 2A,B; Table S2). The adsorption of AMP presented the same trend when adsorbed onto rutile,^49^ clays,^43,52–55^ pyrite,^56^ apatite,^57^ iron oxides,^51,58^ calcium sulfate,^59^ and allophane.^48^ For zirconium oxide, the maximum adsorption occurred at neutral pH, when the pH was acidic or basic the adsorption decreased.^60^ However, when dissolving AMP in artificial seawater-B (high Ca^2+^ and Cl^–^ concentrations) the adsorption cannot be explained as only due to electrostatic interactions (Figure 2C, Table S2), so the effect of the salts in the artificial seawater should be taken into account.

### 4.2 Adsorption Isotherm of the Molecules

All data of this work were fitted using nonlinear isotherm models (Table 1), because they produced more reliable results than linear models.^30,31,61,62^

For adenine, in the current work the SIPs isotherm model presented slightly better *R* ^2^/RMSE parameters than the Langmuir model (Table 1). Furthermore, the adsorption of adenine onto Na^+^/Fe^3+^/Cu^2+^-montmorillonite fitted well in the nonlinear SIPs isotherm model.^26^ For other works, the Langmuir isotherm model presented the best fit.^63–66^ However, the data of this work were fitted in nonlinear isotherm models and the others in linear isotherm models.^63–66^ In addition, the adsorption of adenine onto rutile did not fit into any isotherm model tested.^49^

In the current work, using nonlinear isotherm models, adenosine isotherm fitted well in the Langmuir and SIPs models, with the *R* ^2^/RMSE values of the Langmuir model being slightly better than the SIPs model (Table 1). Adenosine dissolved in water (montmorillonite) or sodium chloride-0.10 mol L^–1^ (rutile) fitted well in the Langmuir isotherm model.^49,66^ However, when dissolving adenosine in artificial seawater (high Mg^2+^ and SO_4_^2–^ concentrations), the best fit was obtained with the Freundlich isotherm model.^66^ This discrepancy could be attributed to the linear isotherm models used.^66^

The nonlinear adsorption isotherms for AMP fitted well in the Langmuir/SIPs models, with the SIPs model presenting slightly better results (Table 1). In addition, the adsorption isotherm model for AMP onto apatite fitted well in the SIPs model.^57^ However, the adsorption of AMP onto mordenite/allophane and montmorillonite/rutile/nontronite/pyrite/iron oxides/zirconium oxide fitted well in the Freundlich isotherm model and Langmuir isotherm model, respectively.^14,48,49,51,53,56,60,66^ Although the adsorption isotherms were obtained for several minerals, the data were not fitted to any mathematical model.^43,54,67^

The SIPS model provided the best fit for the adsorption data, indicating that adsorption sites on montmorillonite exhibit heterogeneous affinities for biomolecules. This suggests that real prebiotic environments might have displayed a range of adsorption capacities depending on mineral composition and ionic conditions. From a prebiotic perspective, this heterogeneity could have influenced the selective accumulation of specific biomolecules, potentially guiding chemical evolution toward biologically relevant compounds.^19,68^

The artificial seawater composition has an effect on the adsorption of adenine, adenosine, and AMP onto montmorillonite. The *q*_max_ values were higher when dissolving these molecules in artificial seawater-B (high Ca^2+^ and Cl^–^ concentrations) (AMP > adenosine > adenine) than in ultrapure water or artificial seawater-A (high Mg^2+^ and SO_4_^2–^ concentrations) (Table 1). These results are unusual, since, for most experiments salts decreased the adsorption of these molecules onto minerals.^26,43,46,47,59,66,67^ However, the role played by salts on the adsorption of nucleic acid bases and related compounds onto minerals is not well understood, since some experiments also show that salts of magnesium and calcium increase the adsorption onto minerals.^51,56^ AMP presented higher adsorption onto pyrite when dissolved in CaCl_2_ (0.075 mol L^–1^) than when dissolved in MgCl_2_ (0.075 mol L^–1^).^56^ In addition, adenosine dissolved in artificial seawater-A (high Mg^2+^ and SO_4_^2–^ concentrations) presented higher adsorption onto montmorillonite than when dissolved in ultrapure water (Table 1).

In the current work, the experiments were performed at pH 5.00 and at this pH montmorillonite has a negative net charge (Table S2). Thus, the adsorption of Ca^2+^ and Mg^2+^ from artificial seawaters onto montmorillonite should occur (eq 9).^23^ Two mechanisms for the adsorption of adenine, adenosine, and AMP onto montmorillonite could be suggested (eqs 10 and 11). It should be noted that according to Lailach et al.^41^ and Sati et al.^69^ the adsorptions of adenine and thymine onto Ca-montmorillonite or Mg-montmorillonite were similar.

$$\mathrm{Mont}+{\mathrm{Ca}}^{2+}/{\mathrm{Mg}}^{2+}=\mathrm{Mont}-{\mathrm{Ca}}^{2+}/{\mathrm{Mg}}^{2+}$$ 9

$$\mathrm{Mont}-{\mathrm{Ca}}^{2+}/{\mathrm{Mg}}^{2+}+\mathrm{molecule}=\mathrm{Mont}-{\mathrm{Ca}}^{2+}/{\mathrm{Mg}}^{2+}-\mathrm{molecule}$$ 10

$$\mathrm{Mont}+{\mathrm{Ca}}^{2+}/{\mathrm{Mg}}^{2+}-\mathrm{molecule}=\mathrm{Mont}-{\mathrm{Ca}}^{2+}/{\mathrm{Mg}}^{2+}-\mathrm{molecule}$$ 11

Mont = montmorillonite, molecule = adenine or adenosine or AMP.

The Ca^2+^/Mg^2+^ cations interact with adenine, the phosphate group of AMP, and probably with the hydroxyl group of the ribose of adenosine.^38,70–72^ However, why did these molecules show the highest adsorption onto montmorillonite when dissolved in artificial seawater-B (high Ca^2+^ and Cl^–^ concentrations)? Since the Ca^2+^ cation (ionic radius 1.00 Å) is larger than the Mg^2+^ cation (0.72 Å),^73^ the solvation sphere of the Ca^2+^ cation is probably smaller than the solvation sphere of Mg^2+^. This could facilitate the interaction between Ca^2+^and these molecules. In addition, because the Ca^2+^ cation is larger than the Mg^2+^ cation, it is more polarizable, so it could more easily bind to adenine, the phosphate group of AMP, and, probably, with the hydroxyl group of the ribose of adenosine than Mg^2+^.

It is possible to highlight these observations using FTIR experiments with adenine samples in different solutions. There are no differences between the FT-IR spectrum of solid adenine and the FT-IR spectrum of adenine lyophilized from ultrapure water solution (Figure 3). However, there are significant differences in the spectra dissolving adenine in different seawaters (Figure 3). Figure 3 presents the FTIR spectra of the lyophilized adenine samples and confirms the observations made by Anizelli et al.^38^ The Ca^2+^ from artificial seawater-B (high Ca^2+^ and Cl^–^ concentrations) coordinates with the amine and imidazole nitrogen and changes the stretching frequencies of the two vibrational modes (Figure 3). It can also be observed that the C=N stretching disappears (Figure 3), because there is a shift in the stretching frequency due to the coordination of the amine, which changes the electronic density around the vicinal carbon–nitrogen double bond. Thus, it is recognized that adenine acts as a bidentate ligand. In the case of the Mg^2+^ from artificial seawater-A (high Mg^2+^ and SO_4_^2–^ concentrations), there is no displacement of the δNH_2_ band (Figure 3). Since magnesium has a smaller radius and greater hydration, it is unable to form a bidentate complex with adenine. Therefore, it coordinates only with the position of greater hardness, which consists of an imidazole nitrogen. It can also be noted that the C=N stretching band is not altered in this situation, indicating that the amine does not coordinate with magnesium (Figure 3). Thermodynamically, calcium is more likely to interact with the adenine, adenosine, and AMP molecules due to its lower hardness and larger size. It was impossible to visualize these changes in the adenosine and AMP spectra because of the large amount of water, even after lyophilization. However, maintaining this reasoning, the insertion of ribose and phosphate would alter the electronic density of the systems and increase the possibilities of coordination with both calcium and magnesium. As calcium is more polarizable depending on the size of the radius, it likely coordinates more efficiently in all systems, especially with AMP, which has more coordination points.

**Figure 3:**
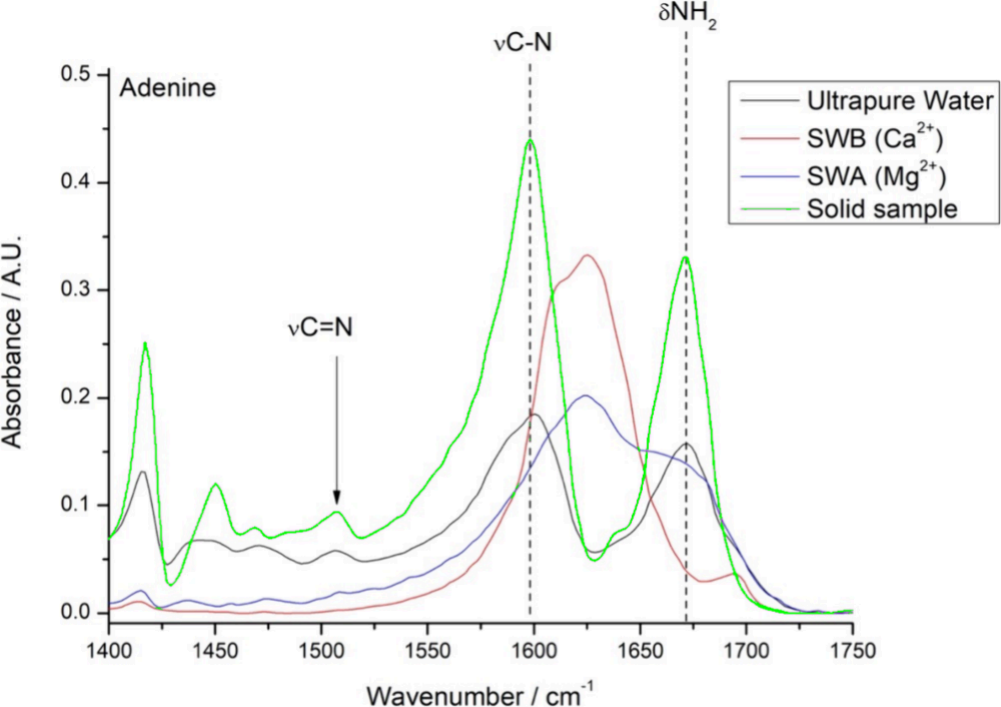
Infrared spectra of lyophilized samples of adenine dissolved in (black line) ultrapure water; (blue line) artificial seawater-A (high Mg^2+^ and SO_4_^2–^ concentrations); (red line) artificial seawater-B (high Ca^2+^ and Cl^–^ concentrations), and for comparison (green line) solid adenine sample. Artificial seawater-A (high Mg^2+^ and SO_4_^2–^ concentrations) and artificial seawater-B (high Ca^2+^ and Cl^–^ concentrations) were prepared as described by Zaia^19^ and Samulewski et al.,^20^ respectively.

In general, heterogeneous systems presented larger *n* values. For the present work, the system heterogeneity could come from the montmorillonite or the molecules (adenine, adenosine, AMP) or even a mix of both.^74^ There are several works showing that the interaction of adenine, adenosine, and AMP with minerals presented an *n* value higher than 1.0, meaning that these systems are heterogeneous.^14,48,57,66,75^ However, in the present work, for adenine, *n* values higher than one (5 experiments) and *n* values lower than 1.0 (4 experiments) were found (Table 1). All experiments of the adsorption of adenosine and AMP, with one exception (adenosine dissolved in artificial seawater-A, at 20 °C), presented *n* values higher than one (Table 1).

For all experiments, the *R*_L_ values for the adsorption of adenine, adenosine, and AMP onto montmorillonite were lower than 1. These values are in good agreement with the Δ*G* values, meaning that the adsorption of these molecules onto montmorillonite is a spontaneous process (Tables 2 and 3).

The Δ*G* values for adenine adsorbed onto montmorillonite and Cu^2+^/Fe^3+^-montmorillonite are in the same range as the present work.^26^ On the other hand, the ΔG value for the adsorption of AMP onto apatite was in the range of −22 kJ mol^–1^,^57^ while in the present work the ΔG values were in the range of −11 kJ mol^–1^ (Table 3). In the current work, all experiments presented positive adsorption entropy, meaning that adenine, adenosine, and AMP in solution are more organized than on the surface of montmorillonite. This is an unexpected result, as usually when a molecule in the liquid phase is adsorbed on the surface of a solid, a reduction in the disorder at the surface onto the solid occurs, since the molecules lose some degree of freedom (rotation, translation), so the entropy is reduced, meaning it should be negative.^76^ One explanation for this apparent discrepancy is that water or artificial seawater salts are forming a huge structure with these molecules in solution. As in the present work, for all experiments with adenine the entropy adsorption was positive.^26^ According to Sowerby et al.^64^ and Pereira et al.^26^ for adenine, the enthalpy adsorption was negative, but our results presented positive values for the adsorption enthalpy (Table 3).

### 4.3 Desorption of the Molecules

At pH 2.0, adenine, adenosine, and AMP were adsorbed onto montmorillonite.^44^ These molecules enter into the interlayers of Na-montmorillonite in the following amounts: adenine 61%, adenosine 71%, and AMP 60%.^44^ For the Perezgasga^44^ experiments (pH 2.0) and our experiments (pH 5.0), adenine, adenosine, and AMP presented the following net charges ++/+, ++/0, and ++/0, respectively (Table S2). Based on the results obtained by Perezgasga et al.^44^ and the data from table S2 we would expect that (a) adenine dissolved in water or in artificial seawaters should enter into the interlayers of montmorillonite because it is positively charged and (b) adenosine and AMP dissolved in water should not enter into the interlayers of montmorillonite, because they are not charged (Table S2), but adenosine and AMP dissolved in artificial seawaters could form complexes with Ca^2+^/Mg^2+^,^70–72^ and they could then enter into the interlayers of montmorillonite.

Adenine and guanine entered into the interlayers of clays, where they bonded to the them very strongly.^25,77^ These molecules could only be withdrawn using a high concentration of sodium hydroxide.^25,77^ At pH 5.0, adenine dissolved in ultrapure water or dissolved in artificial seawater-A (high Mg^2+^ and SO_4_^2–^ concentrations) is positively charged, and thus it is expected that adenine adsorbs strongly to montmorillonite (Table 4). However, dissolving adenine in artificial seawater-B (high Ca^2+^ and Cl^–^ concentrations), approximately 43% of adenine is weakly bonded to the montmorillonite (Table 4). It is probable the complex between Ca^2+^ from artificial seawater-B and adenine did not enter into the interlayers of montmorillonite. The low desorption observed for adenine can be attributed to its adsorption mechanism, involving ion exchange, complexation with metal cations and intercalation of adenine into the interlayers of montmorillonite.^25,66^ In prebiotic scenarios, this suggests that adenine could have been relatively stable in mineral-rich environments, reducing its loss to aqueous phases and potentially increasing its availability for further reactions. This could have favored the accumulation and transformation of nitrogenous bases in early Earth or Mars, contributing to the chemical evolution of prebiotic molecules.

For AMP samples, less than 20% of the molecules were extracted from montmorillonite with Ca^2+^ solution, so AMP is strongly bonded to montmorillonite (Table 4). In addition, AMP could be extracted from montmorillonite only with a solution of NaOH at pH 12.^78^ However, our experiments were performed at pH 5.0 and this pH AMP is a neutral molecule (Table S2). The cations (Ca^2+^/Mg^2+^) from artificial seawaters could form a complex with the phosphate group of AMP, and the complex could enter the interlayers of montmorillonite. For AMP dissolved in ultrapure water the phosphate group could interact with Si–OH, Fe–OH, Al–OH, and Mg–OH oxide sites of montmorillonite.^53^

The adenosine desorption was approximately 40–56% (Table 4), this means that half of the adenosine molecules are weakly bound to the montmorillonite. At pH 5.0, adenosine dissolved in ultrapure water is not charged (Table 4), so it cannot enter into the interlayer of montmorillonite. It is probable that in the case of adenosine dissolved in the artificial seawaters, the ribose caused a steric hindrance, making interaction with cations (Ca^2+^/Mg^2+^) from artificial seawaters difficult.

### 4.4 FT-IR Spectroscopy

The FT-IR spectra data showed that adenine and adenosine interact with montmorillonite in a similar way, meaning, through the NH_2_ group, stretching (C_5_–C_6_), and stretching (C_6_–N_1_) (Figures 1, S2 and Table 5). Although the FT-IR spectrum of adenosine adsorbed onto clay did not show the interaction between glycoside moiety and montmorillonite, this could not be ruled out.^49^ Other authors also observed the same trend in the interaction of adenine with zeolites/montmorillonite/Fe^3+^, Cu^2+^-montmorillonite, and adenosine with montmorillonite.^26,66,79,80^ For adenine and adenosine dissolved in artificial seawater-B (high Ca^2+^ and Cl^–^ concentrations), FT-IR spectra presented a shoulder at 1685 cm^–1^ and lower shift (−10 cm^–1^) of the band due to the NH_2_ group, respectively (Figure S2, Table 5). It should be noted that the Ca^2+^ cation has a larger ionic radius than Mg^2+^ cation, so the interaction with these molecules will be facilitated by its lower hydration.^73^ This interaction probably weakens the interaction of the NH_2_ group with montmorillonite, so approximately 43% of adenine desorbed from montmorillonite (Table 4).

For AMP, the band at 1693 cm^–1^ did not change (Figure S2, Table 5). Thus, the interaction between AMP and montmorillonite could be an outer-sphere surface complex, or the NH_2_ group did not play a role in the adsorption of AMP onto montmorillonite. Because the FT-IR spectrum of montmorillonite presented a broad band at 1010 cm^–1^, changes in the phosphate band of AMP adsorbed onto montmorillonite could not be observed (Figure S2). However, several authors showed that the main interaction between minerals and AMP is through the phosphate group.^54,57,58,60,67,81^ Pedreira-Segade et al.^54^ suggested that at a pH below 4.0, clays interact with nucleotides through their positively charged nucleic base and at a neutral pH through the phosphate group of AMP. In addition, Fornaro et al.^67^ suggested that the interaction between AMP and brucite occurred by a tridentate surface complex with two inner-sphere bonds through the phosphate group and a hydrogen bond with the NH_2_ group of the adenine. An inner-sphere bond between montmorillonite and the phosphate group of AMP could explain why only less than 20% of AMP was desorbed from montmorillonite (Table 4). Raman spectra of AMP adsorbed onto apatite presented changes in the bands due to the adenine ring breathing mode, in-plane adenine ring mode, and adenine phosphate group.^57^

## 5 Relevance of These Seawater Solutions to Prebiotic Chemistry

As shown in Table S1, the adsorption of nucleic acid bases, nucleosides, and nucleotides has been studied using several minerals. Furthermore, this table demonstrates that for most of these works the molecules were dissolved in water (Table S1, see in red) or sodium chloride solutions (Table S1, see in blue). As discussed elsewhere, water or sodium chloride are not models for the seas of the Earth before the origin of life.^19^ Sodium chloride and magnesium chloride mixtures have also been used as models for seawater in some studies (Table S1, see in green). These mixtures, indeed, resemble the composition of today’s seawater.^82^ Our group has also published 5 works using an artificial seawater that resembles today’s seawater,^82,83^ with high Na^+^ and Cl^–^ concentrations^45,46,79,83,84^ (Table S1). Some experiments were carried out using buffers (Table S1, see in green), however, they did not exist or, if they existed they would probably have been in very low concentrations, in the seas of the prebiotic Earth (Table S1).

Using hot water, Izawa et al.^21^ extracted cations and anions from meteorite samples of the Tagish Lake. From the cation and anion concentrations obtained by Izawa et al.,^21^ we suggested the artificial seawater-A (high Mg^2+^ and SO_4_^2–^ concentrations).^19^ This seawater was used in the present work, as well as in 4 other works published by our group (Table S1).^26,47,66,80^ However, there are some doubts about a terrestrial ocean with high Mg^2+^ and SO_4_^2–^ concentrations before the origin of life, even if these ions were delivered to Earth by meteorites, because, due to the widespread seafloor weathering, SO_4_^2–^ could be reduced by reactions in a Fe^2+^-rich seawater and Mg^2+^ could be incorporated Mg-rich secondary minerals.^85,86^ Nevertheless, this seawater could be a model for Mars and other moons in the solar system.^87–89^

Based on the work of Halevy and Bachan^22^ our group suggested an artificial seawater with high Ca^2+^ and Cl^–^ concentrations.^20^ This seawater was named artificial seawater-B (high Ca^2+^ and Cl^–^ concentrations), and was used in the current work as well as in 2 other works.^20,90^ It should be noted that the salts of these seawaters had an effect on how thiocyanate and ferrocyanide adsorbed onto minerals.^20,90^ This seawater contains only Cl^–^ as an anion, it does not contain SO_4_^2–^, because this anion only appeared in seawater when the oxygen increased.^91^ In addition, the concentration of the cations of this seawater has the following order of magnitude: Ca^2+^ ≫ K^+^ ≈ Na^+^ > Mg^2+^.^20^ This seawater with high Ca^2+^ and Cl^–^ concentrations is very different from those presented in Table S1. The composition and proportion of salts in this seawater, probably best represent the salts present in the prebiotic Earth’s ocean.

Early Mars is hypothesized to have had transient bodies of liquid water with varying salinity and ionic compositions. The presence of sulfates and magnesium-rich minerals, as suggested by in situ analyses from Martian rovers,^92,93^ supports the plausibility of seawater-like conditions in some Martian environments. In particular, the high Mg^2+^ and SO_4_^2–^ content of Seawater-A resembles the brines found in Martian regolith, making it a relevant model for adsorption studies in Martian analog conditions. The role of such saline environments in concentrating organic molecules is crucial for understanding the plausibility of prebiotic chemistry on Mars.

The current work presents two important results: (1) adenine, adenosine, and AMP dissolved in both seawaters adsorbed onto montmorillonite and (2) the salt composition of the seawater has an effect on the adsorption/interaction of these molecules with montmorillonite. In general, when dissolving adenine, adenosine, and AMP in the water or artificial seawater-A (high Mg^2+^ and SO_4_^2–^ concentrations) the adsorption onto and interaction with montmorillonite were similar. However, a different trend was observed when dissolving these molecules in artificial seawater-B (high Ca^2+^ and Cl^–^ concentrations). The highest adsorption onto montmorillonite was obtained by dissolving these molecules in artificial seawater-B (high Ca^2+^ and Cl^–^ concentrations) in the following order: AMP > adenosine > adenine. Furthermore, the effect of the pH on the adsorption adenine and AMP dissolved in artificial seawater-B (high Ca^2+^ and Cl^–^ concentrations) onto montmorillonite cannot be explained as electrostatic interactions, so other interactions should be considered. However, this is not true when these molecules were dissolved in water or artificial seawater-A (high Mg^2+^ and SO_4_^2–^ concentrations). Another effect of the composition of the salts of the artificial seawater was the adsorption thermodynamics. For all experiments, with one exception, AMP dissolved in artificial seawater-B (high Ca^2+^ and Cl^–^ concentrations), the adsorption was ruled by entropy. In addition, the salts of the seawater have an effect on the desorption of these molecules from montmorillonite. The desorption of adenine dissolved in artificial seawater-B (high Ca^2+^ and Cl^–^ concentrations) was much higher than when dissolved in water or artificial seawater-A (high Mg^2+^ and SO_4_^2–^ concentrations), while an inverse trend was observed for adenosine.

Since artificial seawater-A (high Mg^2+^ and SO_4_^2–^ concentrations) and artificial seawater-B (high Ca^2+^ and Cl^–^ concentrations) could most closely resemble the oceans of Mars/other moons in the solar system and the prebiotic Earth, respectively, further studies using these seawaters should be carried out, with other minerals, such as forsterite, pyrite, iron oxides for the adsorption or polymerization of these molecules or even others (amino acids).

These findings demonstrate that the adsorption of biomolecules onto montmorillonite varies significantly with the ionic composition of the surrounding environment. This has profound implications for prebiotic chemistry, as clay minerals could have acted as concentration sites for organic molecules in both terrestrial and Martian scenarios. The strong adsorption of AMP suggests that phosphate-bearing biomolecules could have been preferentially retained in mineral-rich settings, facilitating their accumulation and potential polymerization into more complex biomolecular structures. This supports the hypothesis that early oceans, lakes, or hydrothermal systems rich in clays could have played a pivotal role in the emergence of life by providing stable microenvironments for biochemical evolution. The presence of sulfate- and magnesium-rich environments on Mars, as inferred from orbital and in situ analyses by the Curiosity and Perseverance rovers, suggests that early Martian aqueous environments could have supported similar adsorption mechanisms to those observed in our experiments.^92,93^ The retention of AMP in montmorillonite under high-salinity conditions is particularly relevant for potential prebiotic chemistry on Mars. Moreover, studies of icy moons such as Europa and Enceladus have revealed the presence of salt-rich subsurface oceans, which could interact with silicate minerals in ways analogous to our artificial seawater models.^94–98^ These findings suggest that clay minerals could have played a role in concentrating organic molecules in extraterrestrial aqueous environments, a factor that should be considered in future astrobiological exploration missions. These results align with previous studies suggesting that clay minerals could serve as prebiotic molecular concentrators in early planetary environments. The preferential adsorption of phosphate-containing molecules like AMP in high-salinity conditions suggests that early aqueous environments, both on Earth and Mars or even on moons of the solar system, may have selectively retained biomolecular building blocks. This is particularly relevant for astrobiology, as it suggests that adsorption processes may influence the distribution of organic molecules in extraterrestrial aqueous settings.

## 6 Conclusions

When dissolving adenine and AMP in the water or artificial seawater-A (high Mg^2+^ and SO_4_^2–^ concentrations), the effect of the pH on the adsorption onto montmorillonite could be explained by the electrostatic interaction between adenine/adenosine and montmorillonite. However, other interactions than electrostatic should be considered, when adenosine and AMP were dissolved in both artificial seawaters and in artificial seawater-B (high Ca^2+^ and Cl^–^ concentrations), respectively.

For all experiments, nonlinear isotherms fitted well in the Langmuir and SIPs models. Therefore, for adenine and AMP, the SIPs model presented slightly better *R* ^2^/RMSE parameters than the Langmuir model. An inverse trend was observed for adenosine. The highest *q*_max_ values were obtained when dissolving adenine, adenosine, and AMP in artificial seawater-B (high Ca^2+^ and Cl^–^ concentrations). This high adsorption could be due to the interaction between Ca^2+^ and the molecules that favored the adsorption even at high pH. In addition, the following order of adsorption was observed: AMP > adenosine > adenine. In general, the *n* values were higher than 1, meaning the systems (molecules/montmorillonite) are heterogeneous.

For all experiments, *R*_L_ and Δ*G* values were lower than 1 and 0, respectively. Thus, the adsorption of adenine, adenosine, and AMP onto montmorillonite were spontaneous processes, with one exception, for AMP dissolved in artificial seawater-B (high Ca^2+^ and Cl^–^ concentrations), the adsorption of these molecules onto montmorillonite was ruled by entropy.

The experiments of the desorption of the molecules from montmorillonite showed that only less than 20% of AMP dissolved in water or in artificial seawater-A (high Mg^2+^ and SO_4_^2–^ concentrations) or artificial seawater-B (high Ca^2+^ and Cl^–^ concentrations) was desorbed. Furthermore, for adenine dissolved in water or in artificial seawater-A (high Mg^2+^ and SO_4_^2–^ concentrations) less than 20% was desorbed from montmorillonite. However, 40 to 56% of adenosine and 42% of adenine dissolved in artificial seawater-B (high Ca^2+^ and Cl^–^ concentrations) are weakly bound to montmorillonite.

The FT-IR spectra showed that adenine and adenosine interact with montmorillonite through the NH_2_ group, however, for AMP this band did not change. Under the experimental conditions used, the phosphate group of AMP interacts with montmorillonite.

Finally, the selective adsorption and retention of biomolecules in montmorillonite may have implications for biosignature preservation in planetary environments. On Mars, for instance, clay minerals could serve as protective matrices for organic compounds, shielding them from degradation by radiation and oxidation. The differential adsorption of adenine, adenosine, and AMP observed in our experiments highlights potential criteria for identifying prebiotic molecular signatures in future missions searching for biosignatures on Mars and icy moons.

## Supplementary Material



## Data Availability

All data that support the findings of this study are available within the manuscript and the Supporting Information.

## Notes

Adenine, adenosine, and AMP adsorption isotherms and FT-IR spectra of analytes adsorbed onto montmorillonite (Figures S1, S2); review on the adsorption of nucleic acid bases, nucleosides, and nucleotides onto minerals and net charges of montmorillonite and analytes in different pHs (Tables S1 and S2) (PDF)
