# Supplementary material for: Artificial Seawater Models Affect Sorption of Adenine and Related Molecules Sorption onto Montmorillonite: Implications for Early Mars and Earth Oceans
Source: ACS Omega. 2025 Jun 26;10(26):28327–42. doi: 10.1021/acsomega.5c03351 (PMC12242628; doi:10.1021/acsomega.5c03351)
Supplement: Supplementary file 1 [file ao5c03351_si_001.pdf]

Artificial seawater models affect adenine and related molecules sorption onto montmorillonite: implications for early Mars and Earth oceans.

Running head: Seawaters: models for Mars/moons/Earth oceans

Giulio Wilgner Ferreira[1#], Bruno Estevam Pintor[1#], Rafael Block Samulewski[2,*], and Dimas Augusto Morozin Zaia[1,*]

[1]Laboratório de Química Prebiótica-LQP, Departamento de Química, Universidade Estadual de Londrina, CEP 86057-970 Londrina, PR, Brazil

[2]Programa de Pós-Graduação em Ciência e Engenharia de Materiais (PPGCEM) - Universidade Tecnológica Federal do Paraná UTFPR Campus Apucarana CEP 86812-460 Apucarana, PR, Brazil.

[#]GWF and BEP contributed equally to this work

*Correspondence authors: D.A.M.Z. (email: damzaia@uel.br) and R.B.S. (email: blockeness@gmail.com)

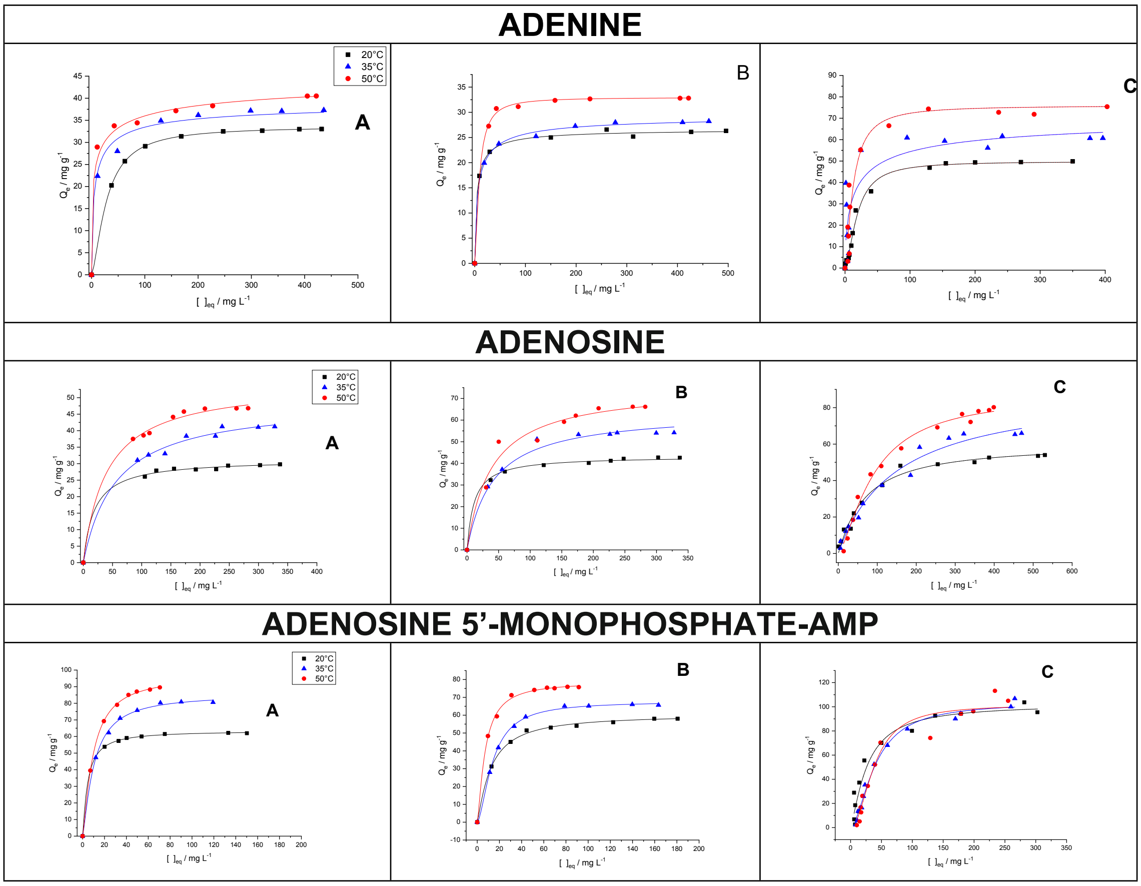

Figure S1: Adsorption isotherms of adenine, adenosine and adenosine 5'-monophosphate adsorbed onto montmorillonite at different temperatures. For all experiments, in Eppendorf tubes, was added 10.0 mg of montmorillonite plus 1.0 mL of adenine, adenosine and adenosine 5'-monophosphate dissolved in: **a)** Ultrapure water, **b)** artificial seawater-A, and **c)** artificial seawater-B. Each value is mean of three experiments. The samples were stirred for 24 h at pH 5.00. Artificial seawater-A (high Mg^{2+} and SO_4^{2-} concentrations) and artificial seawater-B (high Ca^{2+} and Cl^- concentrations) were prepared as described by Zaia[1] (2012) and Samulewski, et. al.[2], (2021), respectively.

ADENINE

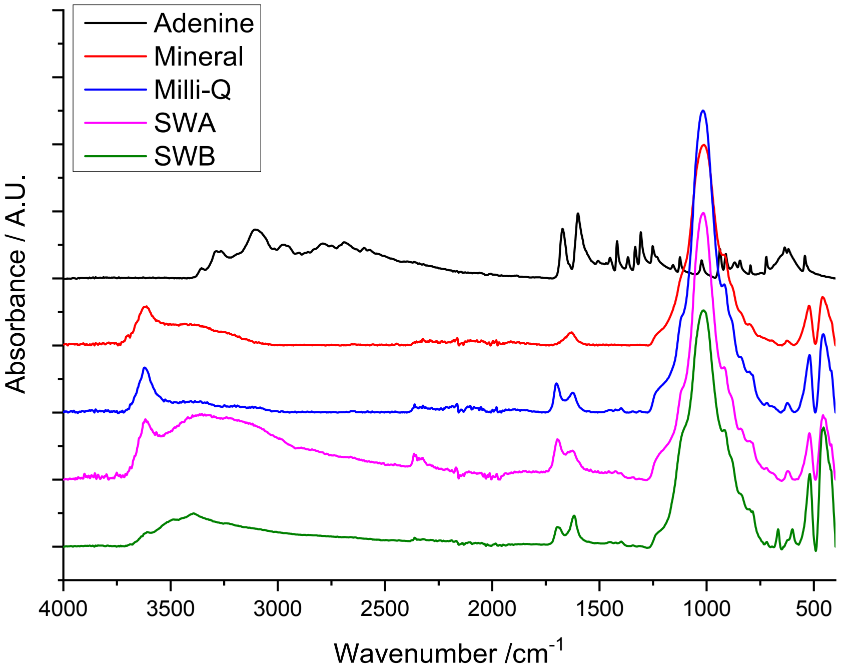

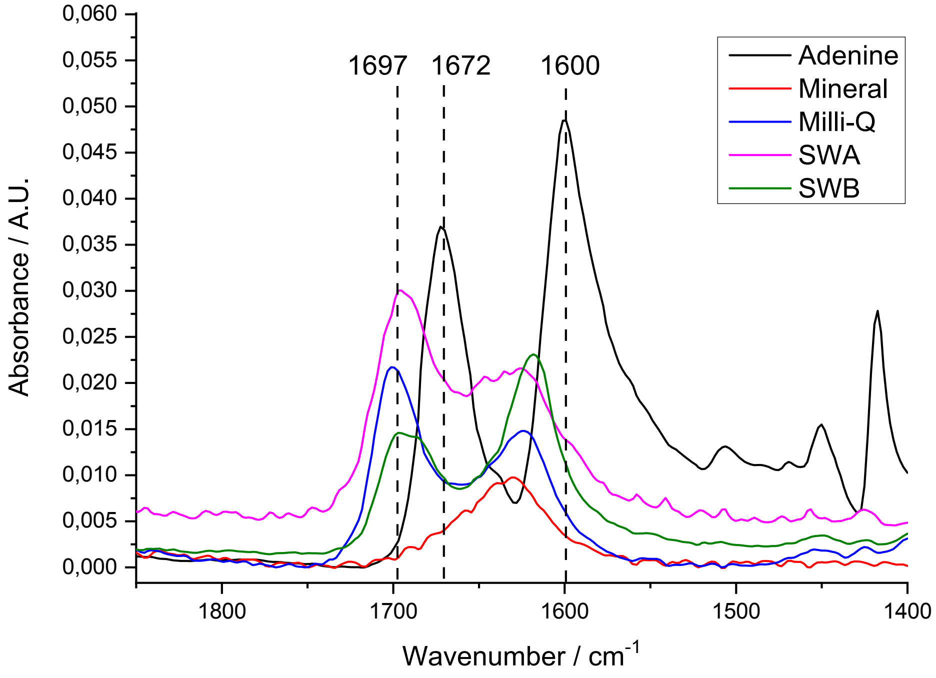

ADENOSINE

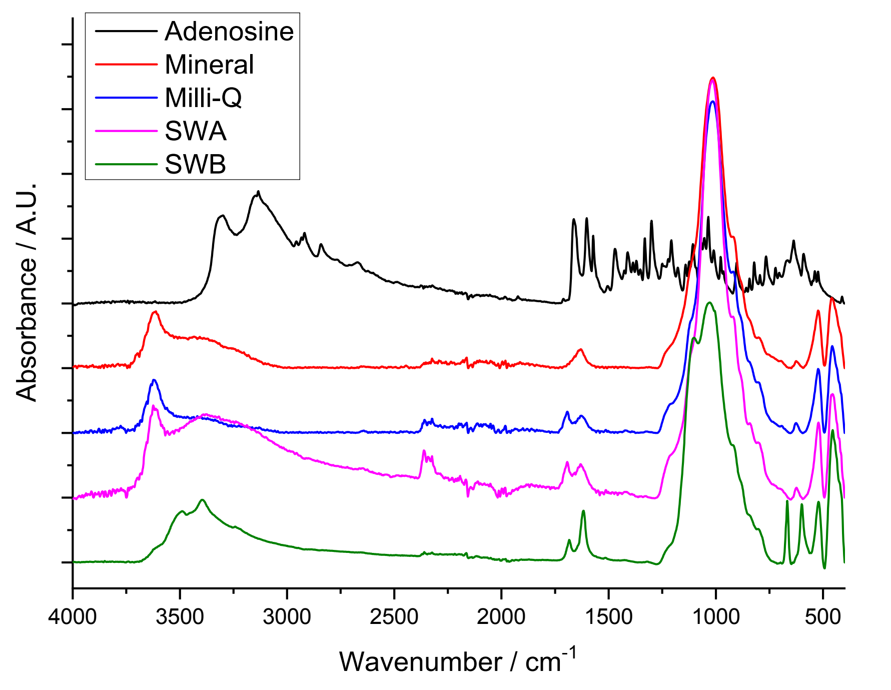

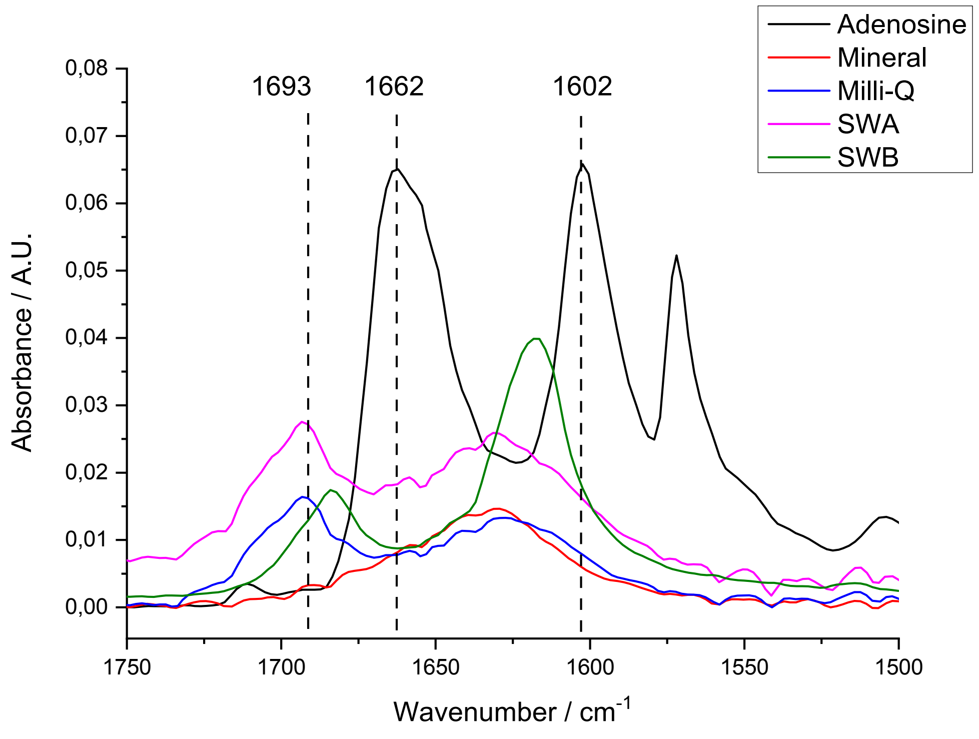

ADENOSINE 5'-MONOPHOPHATE-AMP

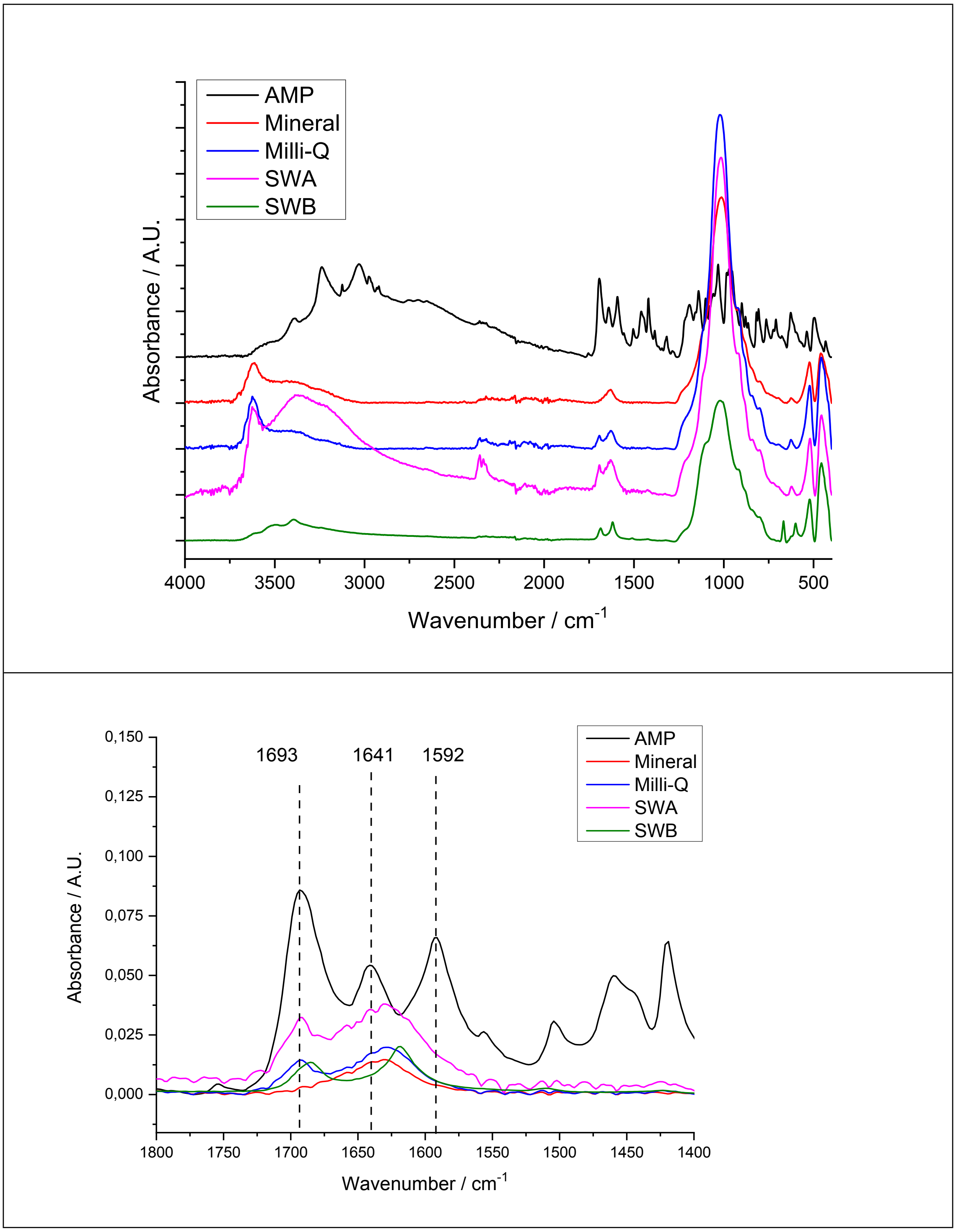

Figure S2: Infrared spectra of lyophilized samples: adenine, adenosine and AMP were dissolved in ultrapure water artificial seawater-A and artificial seawater-B and adsorbed onto montmorillonite. The samples were stirred for 24 h at pH 5.00. Artificial seawater-A (high Mg^{2+} and SO_4^{2-} concentrations) and artificial seawater-B (high Ca^{2+} and Cl^- concentrations) were prepared as described by Zaia[1] (2012) and Samulewski, et. al[2], (2021), respectively.

Table S1: Minerals used for adsorption of nucleic acid bases, nucleosides and nucleotides

Mineral	Experiment	Reference
Akaganéite, goethite, hematite	[C] 5'-AMP, adenosine in pipes buffer (0.2 mol L^{-1}) or $MgCl_2$ (0.075 mol L^{-1}) and NaCl (0.2 mol L^{-1})	Holm et al.[3], 1993
Allophane	[A] Adenine, adenosine, ribose and 5'AMP dissolved in water.	Hashizume and Theng[4], 2007
Aluminum oxide	[A] 5'-AMP, 5'-GMP, 5'-CMP, and 5'-UMP dissolved in water.	Arora and Kamaluddin[5], 2009
Apatite	[B] AMP dissolved in KCl (0.010 mol L^{-1})	Hammami et al[6]., 2015
Bentonite	[D] Alanine, methionine, glutamine, cysteine, aspartic acid, lysine, histidine dissolved in today's artificial seawater	Benetoli et al.[7], 2007
Bentonite, kaolinite, montmorillonite	[D] Adenine, cytosine, uracil and thymine dissolved in today's artificial seawater	Benetoli et al.[8], 2008
Brucite	[B] Uracil, uridine, UMP and AMP dissolved in water and sodium chloride (0.01 and 0.1 mol L^{-1})	Fornaro et al.[9], 2018
Calcium sulfate	[A] AMP and ATP in distilled water	Orenberg et al.[10], 1985
Ferrihydrite	[E] Adenine, thymine and uracil dissolved in artificial seawater rich in Mg^{2+} and SO_4^{2-}	Canhisares-Filho et al.[11], 2015
Graphite (natural)	[A] Adenine, hypoxanthine, thymine, guanine, cytosine, and uracil dissolved in water	Sowerby et al.[12], 2001(a)
Graphite (natural)	[A] Adenine dissolved in water	Sowerby et al.[13], 2001(b)
Magnesium oxide	[A] Adenine, cytosone, hypoxanthine and uracil dissolved in water	Fornaro et. al.[14], 2013
Manganese oxides (MnO, Mn_2O_3, Mn_3O_4, MnO_2)	[A] 5'-AMP, 5'-GMP, 5'-CMP and 5'-UMP dissolved in water.	Bhushan et. al.[15], 2011

Montmorillonite	[A] Pyrimidines, purines and nucleosides dissolved in water	Lailach et al.[16], 1968 Lailach and Brindley[17], 1969
Montmorillonite, kaolinite, $Al(OH)_3$	[A] ATP, ADP, AMP and Gly dissolved in water	Rishpon et al.[18], 1982
M^{n+}-montmorillonite (M^{n+} = Na^{+}, Mn^{2+}, Fe^{3+}, Ni^{2+}, Cu^{2+}, Zn^{2+})	[A] 5'-, 3'-, and 2'-AMP, and 5'-CMP dissolved in water	Lawless et al.[19], 1985
Fe^{3+}, Ca^{2+}-montmorillonite	[A] AMP dissolved in water	Banin et al.[20], 1985
Montmorillonite	[A] Adenine, adenosine dissolved in water	Strašák[21], 1991
Montmorillonite and hydroxylapatite	[F] Adenine, adenosine, 5'-AMP, 5'-ADP and 5'-ATP dissolved in buffer (pipes 0.1 mol L^{-1}), salts (NaCl 0.1 mol L^{-1}, $MgCl_2$ 0.02 mol L^{-1}) at pH 6.7 and artificial seawater at pH 8.6.[&]	Winter and Zubay[22], 1995
Montmorillonite	[A] Adenine, Uracil, adenosine, AMP, ADP, ATP, poly-adenine, poly-uracil dissolved in water with and without HDTMA	Perezgasga et al.[23], 2005
Mg^{2+}-Montmorillonite	[A] Adenine, cytosine, uracil, ribose and phosphate dissolved in water	Hashizume et. al.[24], 2010
Montmorillonite	[A] RNA oligomers dissolved in water	Swadling et. al.[25], 2010
Montmorillonite	[A] Adenine and cytosine dissolved in water	Sciascia et. al.[26], 2011
Sulfide-montmorillonite	[D] Adenine, cytosine, thymine and uracil dissolved in today's artificial seawater	Carneiro et al.[27], 2011

Montmorillonite and nontronite	[C] AMP, CMP, GMP, UMP, dGMP dissolved in NaCl (0.50 mol L^{-1}) and $MgCl_2$ (0.05 mol L^{-1})	Feuillie et. al.[28], 2013
Montmorillonite, nontronite, pyrophyllite, lizardite, chlorite, chrysotile	[C] AMP, CMP, GMP, UMP, dCMP, dGMP, and TMP dissolved in sodium chloride (0.5 mol L^{-1}) and magnesium chloride (0.05 mol L^{-1})	Pedreira-Segade et al.[29], 2016
Na-montmorillonite	[E] Adenine, adenosine and AMP dissolved in artificial seawater rich in Mg^{2+} and $SO_4{}^2$	Villafañe-Barajas et al.[30], 2018
Montmorillonite, nontronite	[B] AMP, adenosine, dAMP, and dGMP dissolved in sodium chloride (0.5 mol L^{-1})	Hao et al.[31], 2019
Fe^{3+}, Cu^{2+}-montmorillonite	[E] Adenine dissolved in artificial seawater rich in Mg^{2+} and $SO_4{}^2$	Pereira et al.[32], 2021
M^{2+}-montmorillonite (M^{2+} = Fe^{2+}, Co^{2+}, Cu^{2+}, Ca^{2+}, Mg^{2+})	[A] Thymine dissolved in water at several pHs	Sati et al.[33], 2022
M^{2+}-montmorillonite (M^{2+} = Fe^{2+}, Cu^{2+}, Ca^{2+}, Mg^{2+})	[C] Cytosine dissolved in buffers (acetic acid/sodium acetate, boric acid/borax)	Bhatt et al.[34], 2022
Prussiam Blue	[A] AMP, GMP, CMP, UMP dissolved in water	Sharma et al.[35], 2016
Olivine, pyrite, calcite, hematite, rutile	[B] ssDNA dissolved in NaCl (0.1 mol L^{-1}) pH 8.1 $KHCO_3$ (0.05 mol L^{-1}) buffer	Cleaves II et. al.[36], 2011
Pyrite and silicon dioxide	[A] Adenine dissolved in water	Plekan et al.[37], 1980
Pyrite	[C] AMP dissolved in sodium acetate (0.2 mol L^{-1}) or artificial seawater (0.075 mol L^{-1} of $MgCl_2$)	Pontes-Buarque et al.[38], 2001
Pyrite, quartz, pyrrhotite, magnetite, forsterite	[A] Adenine dissolved in water	Cohn et al.[39], 2001

Saponite	[A] Purine, adenine, guanine, hypoxanthine, 5’-ADP, 5’-GMP dissolved in water	Weckhuysen et al.[40], 1999
Titanium dioxide (anatase)	[B] Adenine, hypoxanthine, cytosine, uracil, and guanine. As well as: nucleoside, deoxynucleoside, nucleotide, and deoxynucleotide of these bases. All samples were dissolved in sodium chloride (0.10 mol L^{-1})	Cleaves II et al.[41], 2010
Titanium dioxide (anatase)	[A] Adenine, guanine, cytosine, uracil, adenosine, 2’-deoxyadenosine, guanosine, cytidine, 2’-deoxycytidine, and uridine dissolved in water	Vlasova et al.[42], 2015
Titanium dioxide (anatase)	[B] dCMP, dGMP, dAMP, dTMP dissolved in sodium chloride (0.010 mol L^{-1})	Sit et al.[43], 2022
Zeolites (zeolite-A, ZSM-5, zeolite-Y)	[D] Adenine and thymine dissolved today’s artificial seawater	Baú et al.[44], 2012
Zeolite (natural)	[D] Adenine, cytosine, thymine, and uracil dissolved today’s artificial seawater	Anizelli et al.[45], 2015
Zeolite (Fe-ZSM-5 zeolites)	[E] Adenine dissolved in artificial seawater rich in Mg^{2+} and SO_4^{2-}	Anizelli et al.[46], 2016
Zeolite (mordenite)	[A] Adenine adsorption and co adsorption of D-ribose and monophosphate and polymerization dissolved in water	Rodrigues et al.[47] 2022
Zinc ferrite	[A] Ribonucleotides dissolved in water	Iqubal et al.[48], 2015
Zirconia ($ZrOCl_2$)	[A] 5’-AMP, 5’-CMP, 5’-GMP and 5’-UMP dissolved in water	Arora and Kumar[49], 2021

[A] = Experiments were carried out in distilled water; [B] = Experiments were carried out in sodium chloride solutions; [C] = Experiments were carried out using buffers or sodium chloride and magnesium chloride; [D] Today's artificial seawater (Bearman et al.[50], 2004), the following salts were dissolved 1.0 L of water: NaCl (28.57 g), $MgCl_2$ 6H2O (3.88 g), KBr (0.103 g), $CaSO_4$ (1.308 g), K_2SO_4 (0.832 g), H_3BO_3 (0.028 g), and $MgSO_4$ (1.787 g); [E] Artificial seawater rich in Mg^{2+} and SO_4^2 suggested by Zaia (2012) based on the work of Izawa et al.[51] (2010). The following salts were dissolved 1.0 L of water: Na_2SO_4 (0.271 g), $MgCl_2.6H_2O$ (0.500 g), $CaCl_2$ $2H_2O$ (2.50 g), KBr (0.050 g), K_2SO_4 (0.400 g), and $MgSO_4$ (15.00 g); [F] Artificial seawater used by Winter and Zubay[52], (1995), the following salts were dissolved 1.0 L of water: NaCl (27,17 g), $MgCl_2$ (23,78 g), $MgSO_4$ (14,44 g) K_2SO_4 (0.854 g), and Na_2CO_3, (0.127 g). / aAMP = adenosine 5'-monophosphate, ADP = adenosine 5'-diphosphate, ATP = adenosine 5'-triphosphate, CMP = cytidine 5′-monophosphate, dCMP = 2′-deoxycytidine-5′-monophosphate, dDGMP = Deoxyguanosine, GMP = guanosine 5′-monophosphate, HDTMA = hexadecyltrimethyl ammonium bromide, PIPES = piperazine-N,N'-bis(2-ethanesulfonic acid), ssDNA = single-stranded deoxyribonucleic acid, TMP = thymidine, UMP = uridine 5′-monophosphate,

Table S2: Net charges of montmorillonite, adenine, adenosine, and adenosine 5'-monophosphate in different pHs

pH	Net charge (*analyte/**montmorillonite**)		
	Adenine	Adenosine	adenosine 5'-monophophate-AMP
2.0	++/-	++/-	++/-
3.0	++/-	++/-	++/
4.0	++/	+/-	+/-
5.0	+/-	0/-	0/-
6.0	0/-	0/-	0/-
7.0	0/	0/-	-/-
8.0	0/-	0-	-/-
9.0	_/-	0-	--/
10.0	--/-	0/-	--/-
11.0	--/-	0/-	--/-

*The net charges of molecules in different pHs were based on the pKa values of them: adenine pKa 4.83 and 9.83, adenosine pKa 3.6 and 12.4 and adenosine 5'-monophosphate-AMP 3.8 and 6.2 (Haynes, 2016-2017). **The net charge of montmorillonite was based on the value of the point of zero charge (pH_{PZC}) of it. The pH_{PZC} montmorillonite was determined as described by Uehara[53]# (1979). The pHpzc of montmorillonite = 1.10 ± 0.03 (n = 10)

REFERENCES:

(1) Zaia, D. A. M. Adsorption of amino acids and nucleic acid bases onto minerals: a few suggestions for prebiotic chemistry experiments. *Int. J. Astrobiol.* **2012**, *11*, 229-234. http://dx.doi.org/10.1017/s1473550412000195

(2) Samulewski, R. B.; Pintor, B. E.; Ivashita, F. F.; Paesano Jr., A.; Zaia, D. A. M. Study of Ferrocyanide Adsorption onto Different Minerals as Prebiotic Chemistry Assays. *Astrobiology* **2021**, *9*, 1121-1136. http://dx.doi.org/10.1089/ast.2020.2322

(3) Holm, N. G.; Gözen, E.; Ferris, J. P. Polynucleotides on iron oxide hydroxide polymorphs. *Origins Life Evol. Biosphere* **1993**, *23*, 195-215. https://doi.org/10.1007/BF01581839

(4) Hashizume, H.; Theng, B. K. G. Adenine, adenosine, ribose and 5'-AMP adsorptions to allophane. *Clays Clay Miner.* **2007**, *55*, 599-605. https://doi.org/10.1346/CCMN.2007.0550607

(5) Arora, A. K.; Kamaluddin, Role of Metal Oxides in Chemical Evolution: Interaction of Ribose Nucleotides with Alumina. *Astrobiology* **2009**, *9*, 165-171. https://doi.org/10.1089/ast.2007.0143

(6) Hammami, K.; El Feki, H.; Marsan, O.; Drouet, C. Adsorption of nucleotides on biomimetric apatite: the case of adenosine 5' monophosphate (AMP). *Appl. Surf. Sci.* **2015**, *353*, 165-172. http://dx.doi.org/10.1016/j.apsusc.2015.06.068

(7) Benetoli, L. O. B.; de Souza, C. M. D.; da Silva, Kl. L.; de Souza Jr, I. G.; de Santana, H.; Paesano Jr, A.; da Costa, A. C. S.; Zaia, C. T. B. V.; Zaia, D. A. M.; Amino Acid Interaction with and Adsorption on Clays: FT-IR and Mössbauer Spectroscopy and X-ray Diffractometry Investigations. *Origins Life Evol Biospheres* **2007**, *37*, 479–493 DOI https://doi.org/10.1007/s11084-007-9072-7

(8) Benetoli, L. O. B.; de Santana, H.; Zaia, C. T. B. V.; Zaia, D. A. M. Adsorption of nucleic acid bases on clays: an investigation using Langmuir and Freundlich isotherms and FT-IR spectroscopy. *Monatsh. Chem.* **2008**, *139*, 753-761. https://doi.org/10.1007/s00706-008-0862-z

(9) Fornaro, T.; Brucato, J. R.; Feuillie, C.; Sverjensky, D. A.; Hazen, R. M., Brunetto, R.; D'Amore, M.; Barone, V. Binding of nucleic acid components to the serpentinite-hosted hydrothermal mineral brucite. *Astrobiology* **2018,** *18,* 989-1007. https://doi.org/10.1089/ast.2017.1784

(10) Orenberg, J. B.; Chan, S.; Calderon, J.; Lahav, N. Soluble minerals in chemical evolution: 1. Adsorption of 5'-AMP on $CaSO_4$ a model System. *Origins Life* **1985**, *15*, 121-129. https://doi.org/10.1007/BF01809494

(11) Canhisares-Filho, J. E.; Carneiro, C. E. A.; de Santana, H.; Urbano, A.; da Costa, A. C. S.; Zaia, C. T. B. V.; Zaia, D. A. M. Characterization of the adsorption of nucleic acid bases onto ferrihydrite via Fourier transform infrared and surface-enhanced Raman spectroscopy and X-ray diffractometry. *Astrobiology* **2015**, *15*, 728-738. http://dx.doi.org/10.1089/ast.2015.1309

(12) Sowerby, S. J.; Cohn, C. A.; Heckl, W. M.; Holm, N. G. Differential adsorption of nucleic acid bases: relevance to the origin of life. *Proc. Natl. Acad. Sci. U. S. A.* **2001a**, *98*, 820-822. https://doi.org/10.1073/pnas.98.3.820

(13) Sowerby, S. J.; Mörth, C. M.; Holm, N. G. Effect of temperature on the adsorption of adenine. *Astrobiology* **2001b**, *1*, 481-487. https://doi.org/10.1089/153110701753593883

(14) Fornaro, T.; Brucato, J. R.; Branciamore, S.; Pucci, A. Adsorption of nucleic acid bases on magnesium oxide. *Int. J. Astrobiol.* **2013**, *12,* 78-86. https://doi.org/10.1017/S1473550412000444

(15) Bhushan, B.; Shanker, U.; Kamaluddin, Adsorption of Ribose Nucleotides on Manganese Oxides with Varied Mn/O Ratio: Implications for Chemical Evolution. *Origins Life Evol. Biosphere* **2011**, *41*, 469–482 https://doi.org/10.1007/s11084-011-9241-6

(16) Lailach, G. E.; Thompson, T. D.; Brindley, G. W. Adsorption of pyrimidines, purines, and nucleosides by Li-, Na-, Mg-, and Ca-montmorillonite (clay organic studies XII). *Clays Clay Miner.* **1968**, *16*, 285-293. https://doi.org/10.1346/CCMN.1968.0160405

(17) Lailach, G. E.; Brindley, G. W. Specific co-absorption of purines and pyrimidines by montmorillonite (clay-organic studies XV). *Clays Clay Miner.* **1969**, *17*, 95-100.

(18) Rishpon, J.; O'Hara, P.J.; Lahav, N.; Lawless, J. G. Interaction between ATP, metals ions, glycine and several minerals. *J. Mol. Evol.* **1982**, *18,* 179-184. https://doi.org/10.1007/BF01733044

(19) Lawless, J. G.; Banin, A.; Church, F. M.; Mazzurco, J.; Huff, R.; Kao, J.; Cook, A.; Lowe, T.; Orenberg, J. B. pH profile of the adsorption of nucleotides onto montmorillonite. *Origins Life* **1985**, *15*, 77-88. https://doi.org/10.1007/BF01809490

(20) Banin, A.; Lawless, J. G.; Mazzurco, J.; Church, F. M.; Margulies, L.; Orenberg, J. B. pH profile of the adsorption of nucleotides onto montmorillonite. *Origins Life* **1985**, *15*, 89-101. https://doi.org/10.1007/BF01809491

(21) Strašák, M. An unusual reaction of adenine and adenosine on montmorillonite. *Naturwissenschaften* **1991**, *78*, 121-122. https://doi.org/10.1007/BF01131486

(22) Winter, D.; Zubay, G. Binding of adenine and adenine related compounds to the clay montmorillonite and the mineral hydroxylapatite. *Origins Life Evol. Biosphere* **1995**, *25*, 61-81. https://doi.org/10.1007/BF01581574

(23) Perezgasga, L.; Serrato-Díaz, A.; Negrón-Mendoza, A.; de Pablo Galán, L.; Mosqueira, F. G. Sites of adsorption of adenine, uracil, and their corresponding derivates on sodium montmorillonite. *Origins Life Evol. Biospheres* **2005**, *35*, 91-110. https://doi.org/10.1007/s11084-005-0199-0

(24) Hashizume, H.; van der Gaast, S.; Theng, B. K. G. Adsorption of adenine, cytosine, uracil, ribose, and phosphate by Mg-exchanged montmorillonite. *Clay Miner.* **2010**, *45*, 469–475. https://doi.org/10.1180/claymin.2010.045.4.469

(25) Swadling, J. B.; Coveney, P. V.; Greenwell, H. C. Clay minerals mediate folding and regioselective interactions of RNA: a large-scale atomistic simulation study. *J. Am. Chem. Soc.* **2010**, *132*, 13750–13764. https://doi.org/10.1021/ja104106y

(26) Sciascia, L.; Liveri, M. L. T.; Merli, M. Kinetic and equilibrium studies for the adsorption of acid nucleic bases onto K10 montmorillonite. *Appl. Clay Sci.* **2011**, *53*, 657-668. https://doi.org/10.1016/j.clay.2011.05.021

(27) Carneiro, C. E. A.; Berndt, G.; de Souza Junior, I.G.; de Souza. C. M. D.; Paesano Jr, A.; da Costa, A. C. S.; di Mauro, E.; de Santana, H.; Zaia, C. T. B. V.; Zaia, D. A. M. Adsorption of adenine, cytosine, thymine, and uracil on sulfide-modified montmorillonite: FT-IR, Mössbauer and EPR spectroscopy and X-ray diffractometry studies. *Origins Life Evolut. Biospheres* **2011**, *41*, 453–468. http://dx.doi.org/10.1007/s11084-011-9244-3

(28) Feuillie, C.; Daniel, I.; Michot, L. J.; Pedreira-Segade, U. Adsorption of nucleotides onto Fe-Mg-Al rich swelling clays. *Geochim. Cosmochim. Acta* **2013**, *120*, 97–108. http://dx.doi.org/10.1016/j.gca.2013.06.021

(29) Pedreira-Segade, U.; Feuillie, C.; Pelletier, M.; Michot, L. J.; Daniel, I. Adsorption of nucleotides onto ferromagnesian phyllosilicates: significance for the origin of life. *Geochim. Cosmochim. Acta* **2016**, *176*, 81–95. http://dx.doi.org/10.1016/j.gca.2015.12.025

(30) Villafañe-Barajas, S. A.; Baú, J. P. T.; Colín-García, A.; Negrón-Mendoza, A.; Heredia-Barbero, A.; Pi-Puig, T.; Zaia, D.A.M. Salinity effects on the adsorption of nucleic acid compounds on Na-montmorillonite: a prebiotic chemistry experiment. *Origins Life Evol. Biospheres* **2018**, *48*, 181-200. https://doi.org/10.1007/s11084-018-9554-9

(31) Hao, J.; Mokhtari, M.; Pedreira-Segade, U.; Michot, L.J.; Daniel, I. Transition metal enhance the adsorption of nucleotides onto clays: implications for the origin of life. *Earth Space Chem.* **2019**, *3*, 109-119. 10.1021/acsearthspacechem.8b00145

(32) Pereira, R. C.; Teixeira, B. S.; da Costa, A. C. S.; Zaia, D. A. M. Interaction between adenine and Cu^{2+} and Fe^{3+}-montmorillonites: a prebiotic chemistry. *Int. J. Astrobiol.* **2021**, *20*, 223-233. https://doi.org/10.1017/S1473550421000070

(33) Sati, S. C.; Pant, C. K.; Bhatt, P.; Pandey, Y. Thymine adsorption onto cation exchanged montmorillonite clay: role of biogenic divalent metal cations in prebiotic processes of chemical evolution. *Origins Life Evol. Biospheres* **2022**, *52*, 233–247. https://doi.org/10.1007/s11084-022-09633-8

(34) Bhatt, P.; Pant, C. K.; Pandey, P.; Pandey, Y.; Sati, S. C.; Mehata, M. S. desorption of cytosine on prebiotic siliceous clay surface induced with metals dications: relevance to origin of life. *Mater. Chem. Phys. A* **2022**, *291*, 126720. https://doi.org/10.1016/j.matchemphys.2022.126720

(35) Sharma, R.; Iqubal, Md. A.; Kamaluddin, Possible role of Prussian blue nanoparticles in chemical evolution: interaction with ribose nucleotides. *Int. J. Astrobiol.* **2016**, *15*, 17–25. https://dx.doi.org/10.1017/S1473550415000348

(36) Cleaves II, H. J.; Crapster-Pregont, E.; Jonsson, C. M.; Jonsson, C. L.; Sverjensky, D. A.; Hazen, R. A. The adsorption of short single-stranded DNA oligomers to mineral surfaces. *Chemosphere* **2011**, *83*, 1560–1567. https://doi.org/10.1016/j.chemosphere.2011.01.023

(37) Plekan, O.; Feyer, V.; Šutara, F.; Skála, T.; Švec, M.; Cháb, V.; Matolín, V.; Prince, K. C. The adsorption of adenine on mineral surfaces: Iron pyrite and silicon dioxide. *Surf. Sci.* **1980**, *601*, 1973–1980. https://doi.org/10.1016/j.susc.2007.02.032

(38) Pontes-Buarques, M.; Tessis, A. C.; Bonapace, J. A. P.; Monte, M. B. M.; Cortés-Lopez, G.; de Souza-Barros F.; Vieyra, A. Modulation of adenosine 5'-monophosphate adsorption onto aqueous resident pyrite: potential mechanisms for prebiotic reactions. *Origins Life Evol. Biosphere* **2001**, *31*, 343-362. https://doi.org/10.1023/A:1011805332303

(39) Cohn, C. A.; Hansson, T. K.; Larsson, H. S.; Sowerby, S. J.; Holm, N. G. Fate of prebiotic adenine. *Astrobiology* **2001**, *1*: 477-480. https://doi.org/10.1089/153110701753593874

(40) Weckhuysen, B. M.; Leeman, H.; Schoonheydt, R. A Synthesis and spectroscopy of clay intercalated Cu(II) bio-monomer complexes: coordination of Cu(II) with purines and nucleotides. *Phys. Chem. Chem. Phys.* **1999**, *1,* 2875-2880. https://doi.org/10.1039/A901531K

(41) Cleaves II, H. J.; Jonsson, C. M.; Jonsson, C. L.; Sverjensky, D. A.; Hazen, R. M. Adsorption of nucleic acid components on rutile (TiO_2) surfaces. *Astrobiology* **2010**, *10*, 311-323. https://doi.org/10.1089=ast.2009.0397

(42) Vlasova, N. N.; Markitan, O. V.; Golovkova, L. P. Adsorption of components of nucleic acids on a titanium dioxide surface. *Colloid J.* **2015**, *77*, 425-430. https://doi.org/10.1134/S1061933X15040213

(43) Sit, I.; Quirk, E.; Hettiarachchi, E.; Grassian, V. H. Diferential surface interactions and surface templating of nucleotides (dGMP, dCMP, dAMP, and dTMP) on oxide particle surfaces. *Langmuir* **2022**, *49*, 15038-15049. https://doi.org/10.1021/acs.langmuir.2c01604

(44) Baú, J. P. T.; Carneiro, C. E. A.; de Souza Junior, I. G.; de Souza, C. M. D.; da Costa, A. C. S.; di Mauro, E.; Zaia, C. T. B. V.; Coronas, J.; Casado, C.; de Santana, H.; Zaia, D. A. M. Adsorption of adenine and thymine on zeolites: FT-IR and EPR spectroscopy and X-ray diffractometry and SEM studies. *Origins Life Evol. Biospheres* **2012**, *42*, 19-29. https://doi.org/10.1007/s11084-011-9246-1

(45) Anizelli, P. R.; Baú, J. P. T.; Gomes, F. P.; da Costa, A. C. S.; Carneiro, C. E. A.; Zaia, C. T. B. V.; Zaia, D. A. M. A prebiotic chemistry experiment on the adsorption of nucleic acid bases onto a natural zeolite. *Origins Life Evol. Biospheres* **2015**, *45,* 289–306. http://dx.doi.org/10.1007/s11084-015-9401-1

(46) Anizelli, P. R.; Baú, J. P. T.; Valezi, D. F.; Cantom, L. C.; Carneiro, C. E. A.; di Mauro, E.; da Costa, A. C. S.; Galante, D.; Braga, A. H.; Rodrigues, F.; Coronas, J.; Casado-Coterillo, C.; Zaia, C. T. B. V.; Zaia, D. A. M. Adenine

interaction with adsorption on Fe-ZSM-5 zeolites: a prebiotic chemistry study using different techniques. *Microporous Mesoporous Mater.* **2016**, *226*, 493-504. https://doi.org/10.1016/j.micromeso.2016.02.004

(47) Rodrigues, F.; Georgelin, T.; Rigaud, B.; Zhuang, G.; Fonseca, G. F.; Valtchev, V.; Jaber M. Deadlocks of adenine ribonucleotides synthesis: evaluation of adsorption and condensation reactions into zeolite micropore space. *Inorg. Chem. Front.* **2022**, *9*, 4111-4120. https://doi.org/10.1039/d2qi00837h

(48) Iqubal, M.A.; Sharma, R.; Kamaluddin, Studies on interaction of ribonucleotides with zinc ferrite nanoparticles using spectroscopic and microscopic techniques. *Karbala Int. J. Mod. Sci.* **2015**, *1*, 49-59. https://doi.org/10.1016/j.kijoms.2015.06.001

(49) Arora, A. K.; Kumar, P. Prebiotic studies on the interaction of zirconia nanoparticles and ribose nucleotides and their role in chemical evolution. *Int. J. Astrobiol.* **2021**, *20*: 142-149. https://doi.org/10.1017/S1473550421000033

(50) Bearman, G. (Editor); Brown, E.; Colling, A.; Park, D.; Phillips, J.; Rothery, D.; Wright, J. Seawater: its Composition, Properties and Behavior. The Open University, **2004**.

(51) Izawa, M. R. M.; Nesbit, H. W.; MacRae, N. D.; Hoffman, E. L. Composition and evolution of the early oceans: Evidence from Tagish Lake meteorite. *Earth Planet. Sci. Lett.* **2010**, *298*, 443–449. https://doi.org/10.1016/j.epsl.2010.08.026

(52) Winter, D.; Zubay, G. Binding of adenine and adenine related compounds to the clay montmorillonite and the mineral hydroxylapatite. *Origins Life Evol. Biosphere* **1995**, *25*, 61-81. https://doi.org/10.1007/BF01581574

(53) Uehara, G Mineral–Chemical properties of oxisols. In International Soil Classification Workshop, Volume 2; Soil Survey Division-Land Development Department: Bangkok, Thailand, pp. 45–46, **1979.**
